# The genetic architecture of pneumonia susceptibility implicates mucin biology and a relationship with psychiatric illness

**DOI:** 10.1038/s41467-022-31473-3

**Published:** 2022-06-29

**Authors:** William R. Reay, Michael P. Geaghan, 23andMe Research Team, Michelle Agee, Babak Alipanahi, Robert K. Bell, Katarzyna Bryc, Sarah L. Elson, Pierre Fontanillas, Nicholas A. Furlotte, Barry Hicks, David A. Hinds, Karen E. Huber, Ethan M. Jewett, Yunxuan Jiang, Aaron Kleinman, Keng-Han Lin, Nadia K. Litterman, Jey C. McCreight, Matthew H. McIntyre, Kimberly F. McManus, Joanna L. Mountain, Elizabeth S. Noblin, Carrie A. M. Northover, Steven J. Pitts, G. David Poznik, J. Fah Sathirapongsasuti, Janie F. Shelton, Suyash Shringarpure, Chao Tian, Joyce Y. Tung, Vladimir Vacic, Xin Wang, Catherine H. Wilson, Murray J. Cairns

**Affiliations:** 1 School of Biomedical Sciences and Pharmacy, Faculty of Health and Medicine, The University of Newcastle, Callaghan, NSW 2308 Australia; 2 Precision Medicine Program, Hunter Medical Research Institute, Newcastle, NSW 2305 Australia; 3 23andMe, Inc., Sunnyvale, CA 94086 USA

**Keywords:** Genetic association study, Population genetics, Infectious diseases

## Abstract

Pneumonia remains one of the leading causes of death worldwide. In this study, we use genome-wide meta-analysis of lifetime pneumonia diagnosis (*N* = 391,044) to identify four association signals outside of the previously implicated major histocompatibility complex region. Integrative analyses and finemapping of these signals support clinically tractable targets, including the mucin *MUC5AC* and tumour necrosis factor receptor superfamily member *TNFRSF1A*. Moreover, we demonstrate widespread evidence of genetic overlap with pneumonia susceptibility across the human phenome, including particularly significant correlations with psychiatric phenotypes that remain significant after testing differing phenotype definitions for pneumonia or genetically conditioning on smoking behaviour. Finally, we show how polygenic risk could be utilised for precision treatment formulation or drug repurposing through pneumonia risk scores constructed using variants mapped to pathways with known drug targets. In summary, we provide insights into the genetic architecture of pneumonia susceptibility and genetics informed targets for drug development or repositioning.

## Abstract (web summary)

Susceptibility to pneumonia has a genetic component, but specific genes involved remain poorly understood. In this study, genetic signals associated with pneumonia susceptibility are identified, providing information about disease biology and potential targets for treatment.

## Introduction

Pneumonia is characterised as an acute infection of the lung, with fluid filled alveoli and resultant restriction of oxygen intake being a key hallmark of its pathophysiology. There are a number of mechanisms known to cause pneumonia, however, bacterial or viral infection are the most common aetiologies^1^. Pharmacological intervention in pneumonia treatment is largely dependent on the infection source—for instance, bacterial-induced pneumonia is treated with antibiotics. Yearly mortality rates worldwide from pneumonia remain high, even in the developed world where access to antibiotics and routine hospital care is usually unrestricted^2,3^. This necessitates a greater understanding of the mechanisms involved in pneumonia susceptibility and pathogenesis, which could be leveraged to identify novel treatments and inform the repositioning of existing drugs.

There has been considerable work undertaken to identify host factors that influence the onset and clinical course of pneumonia. Twin-based estimates of pneumonia heritability are still lacking, however, the heritability of death due to infections disease has been estimated as high as 40%, although further study is required^4^. There have also been few studies that have used modern statistical genetics approaches to test for the existence of risk-increasing or protective alleles associated with pneumonia with sufficient power for surpassing genome-wide significance. Previously, a genome-wide association study of lifetime self-reported pneumonia diagnosis was published using participants obtained by 23andMe, Inc. that identified a significant signal in the major histocompatibility complex (MHC) region on chromosome six^5^. We sought to increase statistical power to detect association signals by performing a genome-wide meta-analysis of self-reported pneumonia in the 23andMe cohort with SNP effects on a clinically ascertained pneumonia phenotype from the FinnGen consortium.

In this work, we interrogate the genetic architecture of pneumonia to identify association signals that map to clinically relevant biology. We priortise plausible risk genes from these new association signals that implicate a role for processes like mucin function in pneumonia susceptibility. We also estimate genetic correlation with clinically significant phenotypes and find particularly significant correlations with psychiatric disorders and related traits. These data identify prospective targets for clinical intervention and drug repurposing, with support for several plausible opportunities for precision medicine.

## Results

### Common and rare variant loci associated with pneumonia susceptibility

**Fig. 1:**
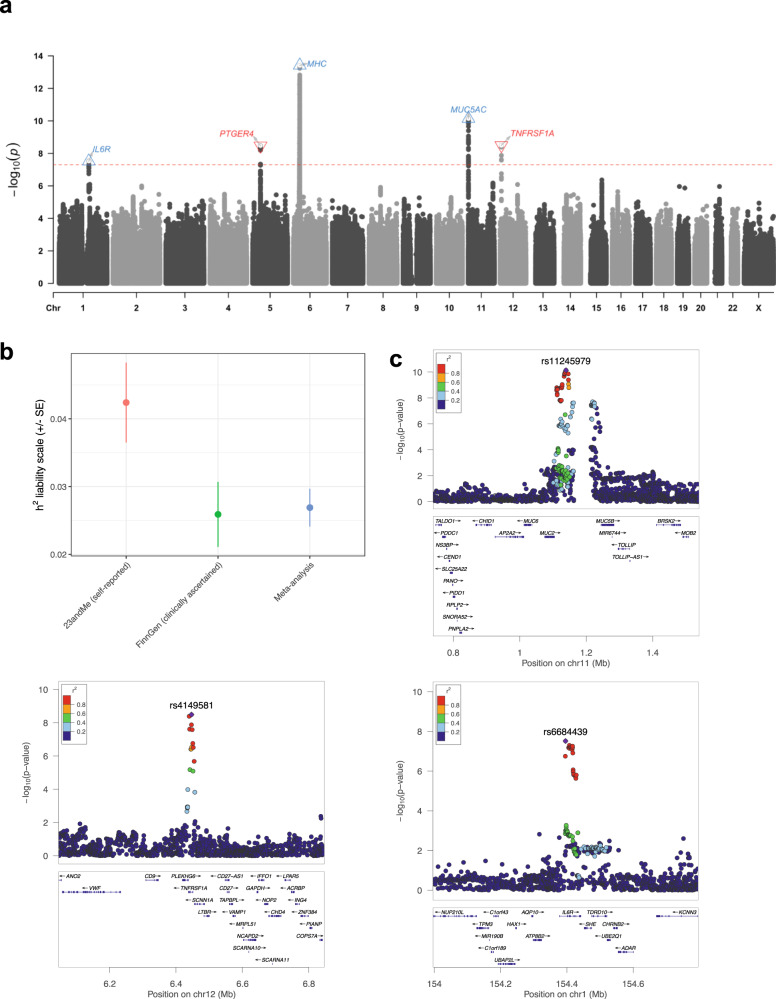
Genome-wide meta-analysis of pneumonia susceptibility. **a** Manhattan plot of common variant GWAS for pneumonia, which was an inverse-variance weighted meta-analysis of SNP-wise log odds estimates of additive association with pneumonia. as is usual practice, each point is the negative log_10_ transformed *P* value of a variant for association with pneumonia, with the red dotted line indicative of genome-wide significance that accounts for multiple comparisons (*P* < 5 × 10^−8^). Closest genes to the lead SNPs are highlighted and labelled on the plot, except for the MHC locus which we denote only as “MHC” due to its complexity. Blue up arrows denote lead SNPs where the minor allele was associated with increased odds of pneumonia, whilst red down arrows denote lead SNPs with protective minor alleles. **b** Estimates of SNP-based heritability (*h*^2^) on the liability scale for the 23andMe and FinnGen cohorts individually, as well as the using the inverse-variance weighted effects meta-analysis of the two cohorts. The error bars represent the standard error of *h*^2^. The sample sizes of the respective GWAS used to calculate heritability were 130,639 for the 23and Me study, 260,405 for FinnGen, and 391,044 for the meta-analysis. **c** Region plots for the three genome-wide significant loci outside of the MHC region in which a gene was mapped to its boundaries. The LD for each variant with the lead SNP, as denoted by the square of the Pearson correlation coefficient between frequencies from the 1000 genomes phase III European reference set, was utilised to colour the points.

We performed a genome-wide meta-analysis of lifetime pneumonia susceptibility (*N*_Cases_ = 74,323, *N*_Controls_ = 316,721, *N*_Effective_ = 240,788) using common and rare (MAF < 0.01) overlapping variants from 23andMe and FinnGen release six, with 6,896,087 and 882,363 common and low frequency sites tested, respectively. The bivariate genetic correlation estimate (*r*_*g*_) between the input self-reported pneumonia 23andMe GWAS and the clinically ascertained pneumonia phenotype definition utilised in the FinnGen data was high and significantly non-zero (*r*_*g*_ = 0.71, SE = 0.12, *P* = 5.72 × 10^−9^). This supports the pooling of these two acquisition methods of the pneumonia phenotype, although we explicitly tested heterogeneity between phenotypes for all SNPs genome-wide (Supplementary Fig. 1). We estimated the SNP-based heritability (*h*^2^_SNP_) as approximately 2.69% on the liability scale (Fig. 1b) using linkage disequilibrium score regression (LDSR)^6^, with the prevalence of pneumonia in the FinnGen release 6 cohort of 12.96% set as the population prevalence for liability scale conversion, however, we acknowledge the population prevalence of pneumonia is difficult to quantify. As a result, we re-estimated *h*^2^ using a more conservative population prevalence value based on phenotype data from the UK biobank (3.20%), resulting in a lower liability scale estimate of *h*^2^_SNP_ = 0.0176. The point estimate of SNP-based *h*^2^ was higher in the 23andMe cohort with the self-reported phenotype, *h*^2^_SNP_ = 0.0424, but the estimate was more precise in the meta-analysis than in either 23andMe or FinnGen alone: *Z*_Meta_ = 9.61, *Z*_23andMe_ = 7.19, and *Z*_FinnGen_ = 5.40. In line with previous comparisons between self-reported and clinically ascertained phenotypes, the heritability estimate was lower in FinnGen than 23andMe. There was some evidence of test statistic inflation when visualised as a quantile-quantile plot (Supplementary Fig. 2), however, the proportion of the polygenic signal (mean *χ*^2^ inflation) in the meta-analysis attributed to model misspecification and/or confounding as indexed by the LDSR intercept was still a modest value of around 17% (SE = 5.3%), given LDSR ratios between 10 and 20% are not uncommon in large GWAS^6^. The mean *χ*^2^ of 1.14 was also large enough to estimate heritability.

There were five common genomic loci that surpassed the conventional genome-wide significance threshold (*P* < 5 × 10^−8^, Table 1, Fig. 1a, c). All the lead SNPs for these loci remained genome-wide significant upon conservatively correcting the *P* values for the LDSR intercept. The effect sizes of these common variant signals were small in accordance with expectation, with each SNP increasing or decreasing the odds of pneumonia by around 4–6%. The most significant signal spanned the major histocompatibility complex (MHC) region, according with the previously observed association with pneumonia in the 23andMe cohort^5^. Due to the complexity of this region, we define the MHC signal as a single locus, with the minor allele of the lead common SNP associated with a small increase in the odds of pneumonia (rs9275211-C allele: OR = 1.06 [95% CI: 1.05, 1.08], *P* = 3.83 × 10^−14^).

**Table 1:** Lead SNPs within common genome-wide significant loci associated with pneumonia.

Lead SNP	Locus	EA/NEA	EAF (NFE)	EAF (FIN)	OR	95% CI	*P*_GWAS_	*P*_Het_
rs9275211	MHC	C/T	0.18	0.16	1.06	1.05, 1.08	3.83e^−14^	0.83
rs11245979	chr11:1110395-1225078	C/T	0.31	0.38	1.05	1.03, 1.06	7.25e^−11^	0.60
rs4149581	chr12:6440009-6455098	C/T	0.42	0.42	0.96	0.95, 0.98	3.22e^−9^	0.60
rs9283753	chr5:40486896-40524860	T/C	0.57	0.50	1.04	1.03, 1.05	3.39e^−9^	0.43
rs6684439	chr1:154395212-154428283	T/C	0.37	0.30	1.04	1.03, 1.05	3.05e^−8^	0.96

Common (MAF > 0.01) lead SNPs were defined as independent SNPs (*r*^2^ < 0.1) within each genomic locus. The effect allele (EA) and non-effect allele (NEA) was reported for this table such that the effect allele was the minor allele. The effect allele frequency (EAF) is denoted in gnomAD v2.1.1 for non-Finish Europeans (NFE) and Finns (FIN). All odds ratio and their respective confidence intervals were calculated relative to the EA. Heterogeneity of effect between the 23andMe and FinnGen cohorts was tested using Cochran’s *Q*, with the *P* value of that test reported here (*P*_Het_). Due to the complexity of locus 1 (MHC), we report only a single common SNP for this locus. Locus coordinates in hg19 assembly.

The most significant common frequency signal outside of the MHC in this study was a region located on chromosome 11, with the lead SNP (rs11245979) located upstream of the *MUC5AC* gene that encodes a mucin protein, with three other genes that encode a mucin protein within 400 kilobases of the lead SNP (*MUC6*, *MUC2*, and *MUC5B*). Importantly, rs11245979 was similarly associated in the 23andMe (*P* = 2.98 × 10^−6^) and FinnGen (*P* = 5.03 × 10^−6^) cohorts, and thus, there was no appreciable evidence for differences in population structure between the input GWAS driving this signal. Mucins are heavily glycosylated proteins that play a number of important roles, particularly in relation to the maintenance of mucosal barriers^7^. Mucin genes are also known the exhibit somewhat pervasive genomic complexity and evidence of heterogeneity between populations, thus, to ensure that this signal is not just an artefact of this, we performed a phenome-wide association study (pheWAS) of the lead SNP in the IEUGWAS database and found that this variant was associated with predominantly respiratory phenotypes relevant to pneumonia. Specifically, it was linked to asthma, self-reported regular cough and mucus, and eosinophil count at a conventional phenome-wide significance threshold (*P* < 1 × 10^−5^, Supplementary Data 1). Given rs11245979 was associated with asthma at a more stringent level of genome-wide significance, we performed colocalisation analyses and found strong evidence that this region was associated with both traits but there were two distinct causal variants in this region (posterior probability = 80.3%). We caution that this assumes the existence of a single causal variant, which may be unrealistic given the complexity of this region. As we visualise in Supplementary Fig. 3, if one utilises a less conservative prior probability of a shared causal variant than there is some evidence that there is a shared underlying causal variant with the posterior probability for that hypothesis rising over 60%. It also should be noted that the association profile of this variant may change as more GWAS emerge, and thus, the phenome-wide association profile requires ongoing investigation.

The third most significant locus was largely physically mapped within the bounds the gene encoding Tumour Necrosis Factor Receptor Superfamily, Member 1A (*TNFRSF1A*). A pheWAS of the lead SNP for this locus also revealed strong associations with inflammatory phenotypes like white blood cell counts, multiple sclerosis, and ankylosing spondylitis (Supplementary Data 2). An intergenic region was the fourth non-MHC locus, with the closest gene to the lead SNP encoding the prostaglandin E receptor 4 (*PTGER4*) and pheWAS revealing this SNP as strongly associated with Crohn’s disease and allergic rhinitis (Supplementary Data 3). Finally, the last genome-wide significant signal uncovered in this study in this study is encompassed within the gene encoding the alpha subunit of the interleukin-6 receptor (*IL6R*) and the lead SNP exhibits a phenome-wide association profile consistent with the complex role of IL-6 in the immune system and other processes (Supplementary Data 4). Importantly, despite the meta-analysis combining a self-reported phenotype with clinically ascertained pneumonia diagnoses, no lead SNP in the demonstrated even nominal heterogeneity in the effect sizes between the two cohorts (Cochran’s *Q*, *P*_Het_ > 0.05, Table 1, Supplementary Fig. 1).

Smoking status was not included as a covariate in the respective GWAS meta-analysed, and thus, we sought to investigate whether genetic variants associated with smoking may confound our findings in this GWAS. Specifically, we genetically conditioned common variant associations on their effect size from a GWAS of smoking initiation and smoking heaviness using mtCOJO^8^. All lead SNPs remained genome-wide significant upon conditioning on either smoking phenotype. Furthermore, SNP heritability was only very marginally impacted by conditioning on these phenotypes (*h*^2^_Conditioned on Cigarettes per day_ = 0.0241, *h*^2^ Conditioned on Smoking initiation = 0.0254). We then sought to replicate our genome-wide significant common loci outside of the MHC using two GWAS from the independent UK Biobank cohort—specifically, we utilised two automated GWAS that encompassed a self-reportedpneumonia phenotype (*N*_Case_ = 6572, *N*_Controls_ = 456,361) and ICD-10 derived pneumonia diagnoses (*N*_Case_ = 10,059, *N*_Controls_ = 398,538). We investigated both phenotyping approaches given our GWAS was a meta-analysis of self-reported and clinically ascertained data. In the self-reported pneumonia UKBB GWAS, we found that no SNPs replicated at genome-wide significance, however, three of the lead SNPs for the chromosome 1, 5, and 11 loci were nominally associated in the same direction (rs6684439: *P* = 7.78 × 10^−3^; rs11245979: *P* = 0.04; rs9283753: *P* = 0.045), The ICD-10 phenotype GWAS in the UKBB did not replicate any of our non-MHC genome-wide significant SNPs at even nominal significance, although there was trend for rs4149581 (*P* = 0.074). It should be noted that a limitation of both of those GWAS is that they focused only on either the self-reported or clinically ascertained phenotype in the UKBB, meaning some controls plausibly would have had pneumonia, and thus, decreasing power. Moreover, the effective sample sizes of these UKBB GWAS were markedly smaller than ours (240,788 in our current discovery meta-analysis versus 25,915 and 39,246, respectively). We also considered two very recent smaller sample-size pneumonia GWAS without publicly available summary statistics to see if we could replicate their findings. Firstly, Chen et al. performed a GWAS of pneumonia susceptibility and severity in the Vanderbilt University Biobank (BioVU, *N*_Case_ = 8889, *N*_Controls_ = 60,767, and *N*_eff_ = 31,019), European ancestry cohort)^9^. They found that a genome-wide significant common signal in Europeans associated with pneumonia severity, with the lead SNP rs10786398 nominally associated in our meta-analysis (*P* = 0.01), whilst we were unable to replicate the significant rare-variant association signal from that study as the variant was not available in our analyses. Moreover, a meta-analysis (*N*_eff_ = 94,584) of a smaller previous FinnGen release (release 2) and ICD-10 derived pneumonia in the UKBB found two genome-wide significant index SNPs in the 15q15.1 region that were directionally consistent in our analyses at a nominal significance threshold: rs76474922 (*P* = 0.025)^10^. The SNP-based heritability estimate from that study also closely mirrored ours (3.3% on the liability scale), supporting the reliability of this study’s estimate in a larger sample, although it should be noted that the samples included in that study partially overlap ours as it used the early second FinnGen release.

Finally, a genome-wide significant association between a rare intergenic variant in the MHC region and pneumonia susceptibility was also uncovered in this study—rs11962863, OR = 1.65 [95% CI: 1.51, 1.79], *P* = 3.94 × 10^−12^. This relatively large effect allele, however, did display statistically significant heterogeneity in its effect between the two cohorts (*P* = 1.4 × 10^−4^). This locus is considerably rarer in the Finnish population (AF = 5.8 × 10^−4^) than non-Finnish Europeans in gnomAD (AF = 2.9 × 10^−3^), which may account for its larger effect size in the FinnGen cohort. Due to the complexity of recombination and linkage in the MHC locus, the functional consequence of this variant remains difficult to interpret at an individual level without considering the local genomic context of affected individuals, such as HLA type. We also detected six additional regions with rare variants that surpassed suggestive significance for association with pneumonia (*P* < 1 × 10^−5^, Supplementary Data 5).

### Integrative gene prioritisation suggests candidate therapeutic targets for pneumonia

**Fig. 2:**
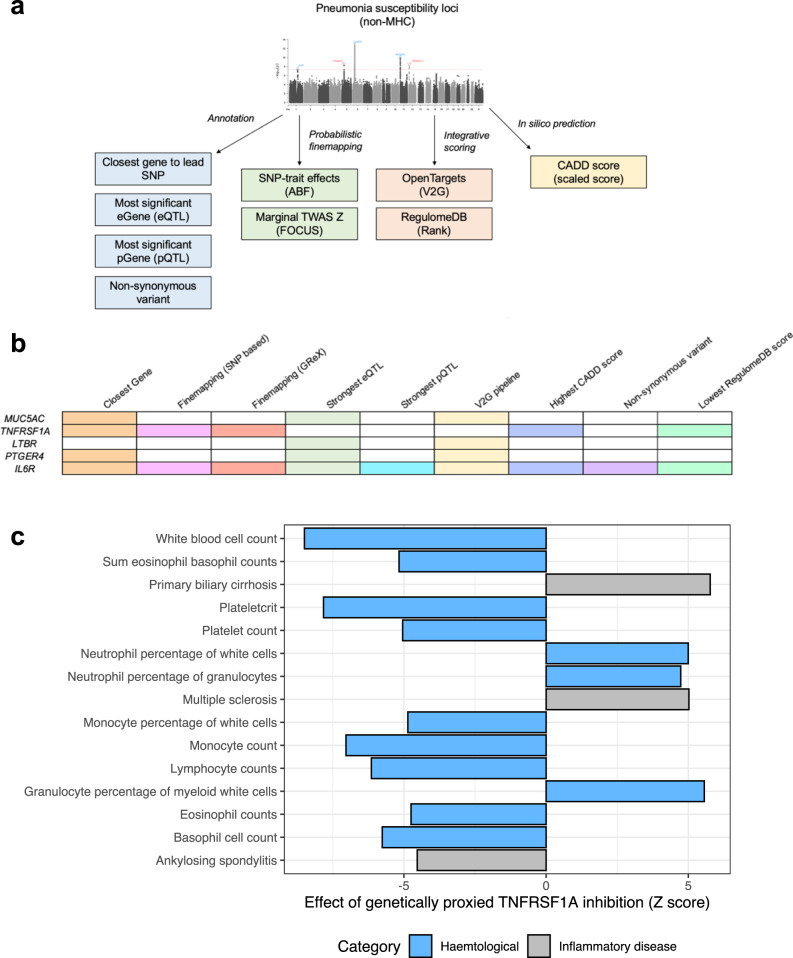
Gene prioritisation for non-MHC pneumonia susceptibility risk genes. **a**, **b** Scoring for gene prioritisation procedure. Each column (**b**) represents a scoring criterion, with a shaded row denoting that said criterion is satisfied for the genes listed by each row. The criteria were as follows: closest gene to lead SNP, gene mapped to 95% credible set for probabilistic finemapping of per variant effect sizes, gene prioritised by probabilistic finemapping of marginal TWAS *Z* (FOCUS), gene annotated with strongest eQTL in the locus, gene annotated with strongest pQTL in the locus, gene prioritised by the OpenTargets *V2G* pipeline, variant annotated to gene with highest CADD score, gene with non-synonymous variant in locus, and variant annotated to gene with lowest RegulomeDB rank score. An unshaded cell for any gene denotes that the gene did not satisfy that criterion. **c** Phenome-wide investigation of genetically proxied *TNFRSF1A* using a blood eQTL with high confidence for annotation as a causal variant. Mendelian randomisation leveraged this eQTL effect size as an IV to estimate the causal effect of *TNFRSF1A* expression on binary and continuous phenotypes in the IEUGWAS database. The results for traits that survive Bonferroni correction are visualised as *Z* scores (MR *β*/MR SE) and have been flipped relative to inhibition of the gene such that *Z* > 0 corresponds to decreased expression increasing the odds (binary) or value (continuous) of the outcome phenotype.

We then subjected each non-MHC common frequency associated locus to an integrative gene prioritisation pipeline (Fig. 2a, b, Online Methods). This approach identified genes from each locus that satisfied the most criteria of the following: closest gene to the lead SNP, gene mapped to variants in the 95% credible set from probabilistic finemapping of SNP effect sizes (Approximate Bayes’ Factors (ABF))^11^, gene with strongest evidence from finemapping (FOCUS) of marginal transcriptome-wide associations study (TWAS) test statistics using models of genetically regulated expression (GReX)^12^, gene with highest score from the *Open Targets Genetics* Variant-to-Gene (V2G) pipeline for the lead SNP^13^, most significant eQTL signal for the locus (eGene), most significant pQTL signal for the locus (pGene), presence of a nonsynonymous variant in the gene, gene with the variant exhibiting the highest Combined Annotation Dependent Depletion (CADD) score^14^, and gene with the variant exhibiting the lowest *RegulomeDB* rank score^15^. The gene *IL6R* satisfied all the above for the locus on chromosome 1, and thus, was likely a causal gene for that signal, although the non-synonymous *IL6R* variant within the bounds of the locus did not quite reach genome-wide significance. *MUC5AC* was the prioritised gene for the chromosome 11 locus by three metrics (closet gene, eGene, and V2G) and the ABF-derived credible set variants were additionally all proximally upstream from that gene. The chromosome 12 locus yielded two plausible genes, *TNFRSF1A* (closest gene, finemapping, CADD score, RegulomeDB score), and *LTBR* (V2G, eGene). Finally, the intergenic signal on chromosome 5 demonstrated some evidence for *PTGER4* (closet gene, V2G, eGene) but the intergenic nature of this locus likely means that further study is warranted to uncover specific biology impacted.

The FOCUS finemapping results, as well as eQTL and pQTL annotated variants, were then investigated to infer a potential direction of expression for each prioritised genes that would be odds increasing for pneumonia. Whilst *MUC5AC* did not have a *cis*-heritable GReX model available for FOCUS, eQTL annotation of individual SNPs consistently suggested that upregulated mRNA expression of *MUC5AC* was associated with elevated odds of pneumonia. Given that *MUC5AC* is mostly very lowly expressed outside of the respiratory epithelium, eQTL estimation remains difficult in bulk tissue, and thus, further study is needed. Moreover, the eQTLs annotated were obtained from the PsychENCODE study of cortical eQTLs, where *MUC5AC* is only lowly expressed. Both FOCUS and eQTL annotation supported that downregulation of *IL6R* would be risk increasing for pneumonia, however, the odds increasing allele of the *IL6R* locus in this GWAS was associated with increased IL-6R protein levels. We caution that the pQTL signal is in high LD with a missense variant (rs2228145), and thus, antigen binding affinity may be altered to create an artefactual pQTL association. These antigen-binding affinity-related effects require further investigation, particularly in light of the phenomenon of the non-synonymous rs2228415-C allele displaying correlation with increased protein abundance from the pQTL study^16^ but with decreased expression via RNAseq derived eQTL, along with decreased C-reactive protein (CRP) levels in the UK biobank, a well-characterised biomarker of IL-6R inhibition. Previous functional analyses of the rs2228145 non-synonymous allele have demonstrated that it likely impairs overall IL-6 signalling as it disturbs the balance between soluble circulating IL-6R and the classical signalling pathway of the membrane bound isoform, and thus, prevents exacerbated IL-6-driven inflammation^17^. This rs2228415-C allele was risk increasing for pneumonia susceptibility at a suggestive significance threshold (*P* = 2.32 × 10^−6^) but has been demonstrated to be protective for disorders like rheumatoid arthritis for which anti-IL-6 receptor agents like tocilizumab are indicated. In summary, it appears from these data that inhibition of *IL6R* would increase the odds of pneumonia, although further study is needed, particularly as this is a GWAS of susceptibility, not pneumonia severity. The remaining three genes, *PTGER4*, *TNFRSF1A*, and *LTBR* displayed mixed directions of expression relative to the pneumonia risk increasing allele when annotating individual SNPs with eQTLs depending on the tissue considered. FOCUS finemapping of marginal TWAS statistics in and around the genome-wide significant locus on chromosome 12 provided further support to *TNFRSF1A* as a pneumonia risk gene, with the highest posterior inclusion probability (*PIP*) in the 90% credible set assigned to a *TNFRSF1A* GReX model in blood for which increased expression was correlated with pneumonia (*PIP* = 0.724). A null model representing potentially untyped genes was in this credible set, however, with very low *PIP* < 2%. FOCUS did not further support *PTGER4* on chromosome 5, with the null model prioritised with the largest *PIP*. Therefore, we consider that there is moderate to strong evidence that *MUC5AC*, *IL6R* and *TNFRSF1A* are involved in the pathophysiology of pneumonia susceptibility. The therapeutic tractability of these targets in the appropriate direction (downregulation of *MUC5AC* and *TNFRSF1A*, upregulation of *IL6R*) was then assessed. Firstly, inhibitors of *MUC5AC* have been in development for oncology, with evidence that compounds like flavonoids, alkaloids, and glycosides may also have *MUC5AC* antagonising properties in the airway^18^. *TNRSF1A* has a structure with ligand available, as well a phase I clinical trial complete for an inhibitor antibody (GSK-1995057) developed for use in acute respiratory distress syndrome^19^. Whilst IL6R inhibitors are available, the clinical utility of the converse, that is, IL6R agonism, requires further consideration due to the complexity of IL-6 biology and likely adverse effects of this mode of action.

To further investigate *TNFRSF1A* inhibition as protective for pneumonia given the existence of an inhibitor antibody with preliminary testing for use in respiratory medicine; a phenome-wide causal estimate (Mendelian randomisation) of genetically proxied *TNFRSF1A* expression was obtained using effect sizes from the IEUGWAS database (MR-PheWAS, Online Methods, Supplementary Data 6 and 7, Fig. 2c). The most significant eQTL for *TNFRSF1A* from the eQTL catalogue also had a high posterior inclusion probability over 95% for that locus (rs1800692, blood tissue), and thus, this SNP was selected as the instrumental variable (IV). After Bonferroni correction (*P*_MR-Wald_ < 6.82 × 10^−6^), we found that genetically proxied *TNFRSF1A* inhibition decreased white blood cell counts (total leucocytes, eosinophils, monocytes, basophils, and lymphocytes), decreased platelet count and was protective for Ankylosing spondylitis. However, inhibition appeared to increase the odds of Multiple sclerosis and Primary biliary cirrhosis, whilst increasing the percentage of neutrophils relative to total leucocyte count. We repeated these analyses with SNP-effect sizes from FinnGen release 6 but no traits survived multiple testing correction. We note that there was a trend (*P*_MR-Wald_ < 1 × 10^−4^) towards a protective effect of *TNFRSF1A* inhibition on FinnGen pneumonia and infection phenotypes, along with evidence for an increased risk of cramps and vitamin B12 anaemia. In summary, *TNFRSF1A* inhibition appears to have a dampening effect on some aspects leucocyte biology and is protective for pneumonia and the autoimmune condition ankylosing spondylitis but conversely displays a risk-increasing relationship with inflammatory-related conditions like Multiple sclerosis, suggesting that more analysis of this candidate therapeutic target is warranted.

### Gene and gene-set association reveal further insights outside of genome-wide significant loci

Common variants were aggregated at gene-level via the MAGMA approach (Supplementary Data 8–10) to boost power for gene discovery^20^. We considered three different strategies to annotate SNPs to genes for gene-based association testing; specifically, mapping SNPs only within the defined coordinates of genes, a conservative extension of genic boundaries to capture regulatory variation (5 kilobase (kb) upstream and 1.5 kb downstream), and a more liberal genic boundary extension (35 kb upstream, 10 kb downstream). *MUC5AC* and *TNFRSF1A* surpassed correction at all three boundary definitions, as did a gene on chromsome 8 outside of the implicated loci from the GWAS, *TOX*, encoding the Thymocyte Selection Associated High Mobility Group Box protein. The *CRP* gene on chromosome 1 also passed strict Bonferroni correction with no boundary extension for SNP annotation. Gene-based results were then utilised to perform competitive gene-set association to identify biological pathways with an enrichment of the pneumonia susceptibility common variant signal relative to all other genes. We calculated a meta-analytic *P* value for each of the 21,765 gene-sets (with at least five overlapping genes) tested across all three boundary definitions value to boost power through leveraging the properties of heavy tail of the Cauchy distribution, such that the covariance between these *P* values derived the same sample is accounted for (Online Methods)^21^. There were no gene-sets that survived multiple-testing correction using a false-discovery rate threshold (FDR) of 5%, however, there were 12 pathways that displayed association using a more lenient FDR < 0.1 threshold (Supplementary Data 11). These gene-sets included heart valve development (*P*_Cauchy_ = 2.49 × 10^−5^, *q* = 0.07), collagen biosynthetic process (*P*_Cauchy_ = 1.86 × 10^−5^, *q* = 0.07), and positive thymic T cell selection (*P*_Cauchy_ = 1.88 × 10^−5^, *q* = 0.07).

A transcriptome-wide association study (TWAS) was then undertaken to identify further genes outside of genome-wide significant loci for which genetically predicted expression was correlated with pneumonia susceptibility^22–24^. We selected SNP weights from three tissues which are plausibly biologically relevant to pneumonia pathophysiology: lung, whole blood, and spleen. After applying Bonferroni correction across all three tissues, which is very conservative given some genes will have significantly *cis*-heritable GReX in multiple tissues, we uncovered three transcriptome-wide significant signals (Supplementary Data 12). Specifically, decreased predicted expression of two proximal genes on chromosome 16 were associated with pneumonia susceptibility, *NPIPB7* in lung (*P*_TWAS_ = 7.18 × 10^−7^) and *SULT1A1* in spleen (*P*_TWAS_ = 8.38 × 10^−7^), whilst upregulated predicted expression of *PSMA4* in whole blood on chromosome 14 also survived correction (*P*_TWAS_ = 9.36 × 10^−7^). Probabilistic finemapping via FOCUS of the marginal TWAS *Z* scores in both implicated regions on chromosome 15 and 16, respectively, was then undertaken using a multi-tissue panel to assess evidence for whether any of these genes are plausible the causal gene in said region. A tissue agonistic finemapping approach, that is, including GReX models based on their most predictive model regardless of tissue, was unable to confidently infer causal genes as all *PIP* were less than 40% and the null model was a member of the 90% credible set, suggesting the potential involvement of a gene without a suitable GReX model. It should be noted that the posterior inclusion probability for the null model to be causal was less than 6% in both instances. However, prioritising whole blood GReX models for the *PSMA4* provided stronger support to this gene (*PIP* = 0.763).

### Pneumonia susceptibility displays significant genetic correlation and partial genetic causality with clinically important phenotypes across the human phenome

We derived genetic correlation estimates between pneumonia susceptibility and 674 UK Biobank (UKBB) GWAS with a trait *h*^2^_SNP_ *Z* score > 4 using LDSR^25^. After Bonferroni correction (*P* < 7.41 × 10^−5^), there were 318 phenotypes that exhibited non-zero genetic correlation with pneumonia susceptibility (Supplementary Data 13). There were several clinically relevant correlations observed in both the positive and negative direction. Traits positively correlated pneumonia susceptibility after correction included anthropometric traits (for example, waist circumference, body mass index, and limb fat mass), psychiatric phenotypes (for example, depression, neuroticism, miserableness), wheezing and chest pain, asthma, angina, diabetes, and chronic obstructive pulmonary disease. Conversely, negative genetic correlations were observed with spirometry measures (lung function), age of last live birth, snoring, ‘never smoked’ status, and paternal age of death.

A latent causal variable (LCV) model was then constructed for the significantly correlated trait-pairs that were uncovered^26^. The LCV approach leverages the bivariate effect size distribution of SNPs in two GWAS and their LD scores to estimate a posterior mean genetic causality proportion (GCP), such that, evidence of partial genetic causality can be distinguished from genetic correlation. We found strong evidence ($\widehat{{|{{{{{\rm{GCP}}}}}}}}|$ > 0.6) for partial genetic causality of five traits on pneumonia susceptibility. Two of these traits related to the gallbladder and gallstones (cholelithiasis): Diagnoses - main ICD10: K80 Cholelithiasis - $\widehat{{{{{{{\rm{GCP}}}}}}}}$ = 0.789, SE = 0.322, *P* = 1.33 × 10^−9^, and Disorders of gallbladder, biliary tract and pancreas - $\widehat{{{{{{{\rm{GCP}}}}}}}}$ = 0.804, SE = 0.145, *P* = 1.80 × 10^−8^; whilst two were measured biochemical traits: Gamma glutamyltransferase (GGT) levels in blood - $\widehat{{{{{{{\rm{GCP}}}}}}}}$ = 0.786, SE = 0.148, *P* = 2.51 × 10^−32^, and CRP levels in blood - $\widehat{{{{{{{\rm{GCP}}}}}}}}$ = 0.705, SE = 0.202, *P* = 1.95 × 10^−4^. The positive sign of the genetic correlation estimates in these four instances, therefore, suggests that gallstones and gallbladder disease, elevated GGT, and elevated CRP would be risk factors for pneumonia. The other trait that exhibited partial genetic causality on pneumonia was the self-reported trait Ever thought that life not worth living - $\widehat{{{{{{{\rm{GCP}}}}}}}}$ = 0.826, SE = 0.142, *P* = 2.45 × 10^−4^. Interestingly, pneumonia susceptibility conversely exhibited partial genetic causality on the psychiatric trait Ever had prolonged feelings of sadness or depression - $\widehat{{{{{{{\rm{GCP}}}}}}}}$ = −0.812, SE = 0.147, *P* = 4.58 × 10^−7^, suggesting a complex bidirectional relationship between pneumonia and affective phenotypes like depression. It is important to emphasise that these posterior mean *GCP* should not be interpreted magnitudes of causal effect and only imply that there is a causal relationship due to the unbalanced nature of the genetic effects^26,27^.

### Strong genetic overlap between pneumonia susceptibility and psychiatric illness within and outside of the major histocompatibility complex region

**Fig. 3:**
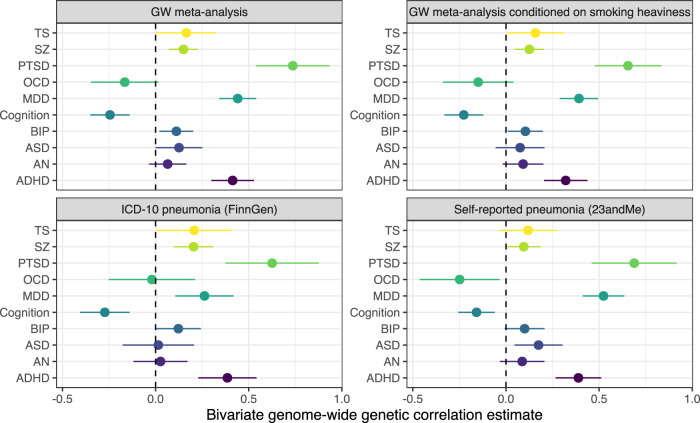
Bivariate genetic correlation estimates between pneumonia susceptibility and psychiatric phenotypes. Genetic correlation was estimated between ten psychiatric GWAS and pneumonia susceptibility GWAS. The forest plot denotes the linkage disequilibrium genetic correlation estimate, whilst the error bars are the 95% confidence internal. The different pneumonia GWAS effect sizes utilised with the panel of psychiatric disorders was as follows: top left – genome-wide meta-analysis of self-reported and clinically ascertained pneumonia; top right – genome-wide meta-analysis of self-reported and clinically ascertained pneumonia genetically conditioned on smoking heaviness (cigarettes per day); bottom left – clinically ascertained pneumonia from FinnGen, and bottom right – self-reported pneumonia from 23andMe. The psychiatric phenotypes were as follows: TS = Tourette’s syndrome (*N* = 14,307), SZ = schizophrenia (130,644), PTSD = post-traumatic stress disorder (*N* = 174659), OCD = obsessive compulsive disorder (*N* = 9725), cognition = general cognitive ability (intelligence, *N* = 269,867), BIP = bipolar disorder (*N* = 51,890), ASD = autism spectrum disorder (*N* = 36,350), AN = anorexia nervosa (*N* = 72,517), and ADHD = attention/deficit-hyperactivity disorder (*N* = 53,293).

Pneumonia susceptibility was found to be robustly genetically correlated with several psychiatric phenotypes from the UKBB cohort. As a result, we investigated whether this signal would remain for psychiatric GWAS with more curated disease phenotypes from the psychiatric genomics consortium (post-traumatic stress disorder (PTSD), attention-deficit/hyperactivity disorder (ADHD), schizophrenia, major depressive disorder (MDD), bipolar disorder, anorexia nervosa, and Tourette’s syndrome)^28–35^, as well as a GWAS of general cognitive function (Online Methods, Fig. 3, Supplementary Data 14)^36^. The pneumonia susceptibility meta-analysis from this study was most significantly positively correlated with MDD (*r*_*g*_ = 0.44, SE = 0.05, *P* = 3.66 × 10^−18^), followed by PTSD (*r*_*g*_ = 0.74, SE = 0.1, *P* = 2.36 × 10^−13^), ADHD (*r*_*g*_ = 0.42, SE = 0.06, *P* = 1.43 × 10^−12^), and schizophrenia (*r*_*g*_ = 0.15, SE = 0.04, *P* = 1 × 10^−4^). General cognitive ability exhibited strong negative correlation with pneumonia susceptibility. We hypothesised that the large magnitude of these correlation estimates could be inflated by heterogeneity in the meta-analysis between self-reported and clinically ascertained pneumonia or an effect of genetic liability to smoking behaviour on both traits. As a result, the self-reported and clinically ascertained pneumonia susceptibility GWAS were utilised separately to estimate genetic correlation with each of the phenotypes. The genetic correlation estimates for PTSD and ADHD remained remarkably stable regardless of which pneumonia phenotype definition was considered (Fig. 3). The MDD genetic correlation was larger with the self-reported pneumonia phenotype (*r*_*g*_ = 0.52) than the clinically ascertained definition (*r*_*g*_ = 0.26) but was significantly non-zero in both instances. Conversely, the correlation estimate was larger for schizophrenia and cognition with clinically ascertained pneumonia. Conditioning SNP-pneumonia effects in the meta-analysis on smoking heaviness (cigarettes per day, mtCOJO analysis) marginally weakened the estimates but all remained statistically significant: MDD (*r*_*g*_ = 0.39), PTSD (*r*_*g*_ = 0.65), ADHD (*r*_*g*_ = 0.32), cognition (*r*_*g*_ = −0.23), and schizophrenia (*r*_*g*_ = 0.13). In summary, there was consistent evidence of genetic correlation between pneumonia susceptibility and psychiatric phenotypes irrespective of the pneumonia phenotype definition and adjustment for smoking related effects. LCV models constructed between each psychiatric GWAS, and the pneumonia susceptibility meta-analysis did not yield a confident estimate of partial genetic causality, further suggesting that complex biological factors may underlie these relationships.

The above models were constructed from SNPs outside of the extended MHC region on chromosome 6, which is an important component of the genetic architecture of pneumonia susceptibility. Thus, we employed the LAVA model to estimate local genetic correlation within the MHC region^37^. The MHC is a difficult region to model in this fashion due to its immense complexity, and all results should, therefore, be interpreted cautiously. The top three most significantly correlated psychiatric GWAS genome wide (MDD, PTSD, and ADHD) were subjected to this analysis, with all three phenotypes and pneumonia susceptibility demonstrating significantly non-zero univariate local heritability in the MHC region. MDD and PTSD demonstrated significant positive local bivariate correlation with pneumonia susceptibility in the MHC region – PTSD: *ρ*_Local_ = 0.68 [95% CI: 0.44, 1], *P* = 1 × 10^−6^; MDD: *ρ*_Local_ = 0.36 [95% CI: 0.16, 0.56], *P* = 4.81 × 10^−4^.

### Genetic interrogation of biochemical targets for pneumonia susceptibility

**Fig. 4:**
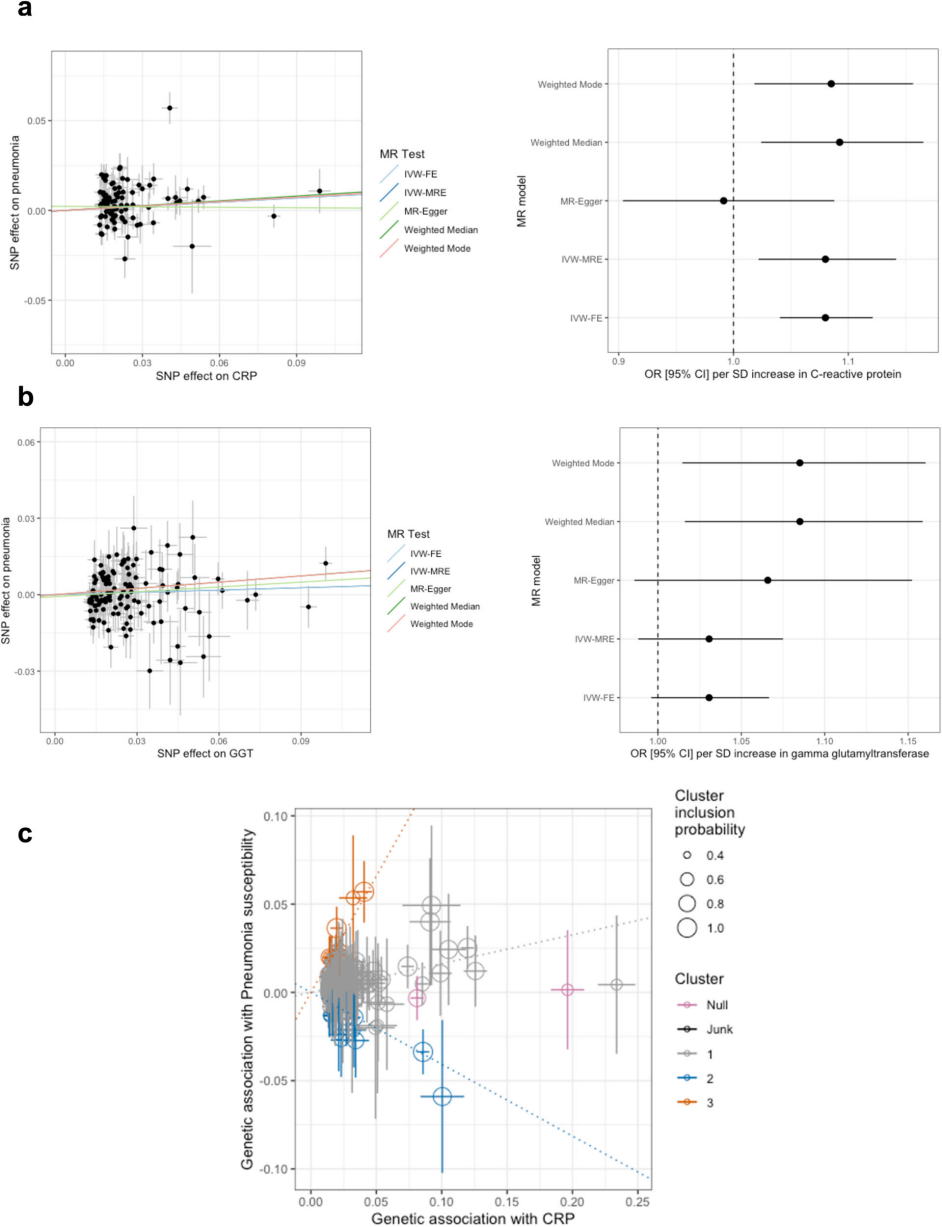
Mendelian randomisation causal estimates of the biochemical traits C-reactive protein and gamma-glutamyltransferase on the odds of pneumonia susceptibility. **a** The left plot is a scatter plot that visualises the effect of each instrumental variable SNP on C-reactive protein (CRP) verses its effect on pneumonia (*N*_CRP_ = 343,524), with the regression trend line the MR estimate from each of the five models implemented. Similarly, the righthand forest plot indicates the pneumonia odds ratio for each of the MR models, with the error bar indicative of the 95% confidence intervals. The MR models were as follows: mode = weighted mode estimator, median = weighted median estimator, IVW-FE = inverse-variance weighted estimator with fixed effects, IVW-MRE = inverse-variance weighted estimator with multiplicative random effects, Egger = MR-Egger regression. **b** The same as (**a**) but relates to gamma-glutamyltransferase (GGT, *N*_GGT_ = 344,104). **c** Output of the MR-Clust approach for the effect of CRP on pneumonia susceptibility using default cluster allocations. This is a mixture model framework that seeks to identify clusters of instrumental variables with similar causal estimates. The size of the point denotes the cluster inclusion probability, relating to the conditional probability of cluster membership. The null cluster, coloured pink, relates to IVs with null effect, whilst the black “junk cluster” are variants that were not parsimoniously assigned to any cluster. The three different non-null or junk clusters are each coloured grey, orange, and blue, respectively, with a trend line indicative of the mean cluster effect. The error bars denote the standard error estimates of the Wald Ratio for each instrumental variable.

We uncovered evidence for non-zero genetic correlation and partial genetic causality between two biochemical indices (GGT and CRP) and pneumonia susceptibility. As a result, Mendelian randomisation (MR) was deployed for these two traits to act as a sensitivity test for the inferred evidence of causality and derive a causal estimate. In contrast to the genome-wide approach of the LCV model, MR leverages specific variants as instrumental variables to proxy the effect of the biochemical trait on the odds of pneumonia susceptibility^38^. We implemented five different MR models with varying underlying assumptions regarding IV validity (Online methods: inverse-variance weighted effect with fixed effects (IVW-FE), inverse variance weighted effect with multiplicative random effects (IVW-MRE), weighted median, weighted mode, and MR-Egger)^38–41^. There estimate per standard deviation (SD) increase in CRP on pneumonia susceptibility was significantly non-zero and positive in all of the models except for the MR-Egger approach (Fig. 4a, Supplementary Data 15); for example, in the IVW-MRE construct OR per SD CRP = 1.08 [95% CI: 1.02, 1.14], *P* = 6.47 × 10^−3^, whilst the IVW-FE produced a more precise positive estimate (*P* = 5.47 × 10^−5^), in line with how multiplicative random effects more explicitly models heterogeneity between IVs. We then considered a series of sensitivity analyses related to heterogeneity and pleiotropy, as well attempting to replicate the CRP → pneumonia susceptibility relationship with an independent CRP GWAS (Online Methods). Firstly, there was evidence of significant heterogeneity between IV estimates (*Q* = 340.30, d*f* = 155, *P* = 6.33 × 10^−16^) and a nominally non-zero MR-Egger intercept (*P* = 0.03), which may indicate confounding pleiotropy. However, given the biological complexity of CRP biology, heterogeneity is not unexpected, and an outlier corrected estimate (MR-PRESSO), was directionally consistent and statistically significant (*P* = 2.84 × 10^−3^). The independent CRP GWAS used as replication demonstrated highly concordant causal estimates across the five methods, with the MR-Egger again the only non-significant test (Supplementary Data 15). Given the evidence of statistical heterogeneity for the effect of CRP, we also applied the MR-Clust model which leverages a mixture modelling approach to detect clusters of IVs with similar causal estimates (Fig. 3c)^42^. This detected one cluster with a mean protective effect of CRP discordant from the overall odds increasing effect using default parameters, as well as also after applying a cluster inclusion probability threshold of 80% (Supplementary Fig. 4). This cluster contains an IV mapped to the IL6R gene which this study indicates has a protective effect on pneumonia susceptibility, supporting proceeding analyses that suggest IL6R inhibition may be odds increasing for pneumonia. Finally, we employed a more conservative MR approach by considering only the effect of a single *cis*-acting IV (rs2794520) mapped to the signal that encompasses the gene which encodes CRP itself. This *cis*-acting model provided more evidence that CRP signalling through the protein itself is odds increasing for pneumonia, even though more complex relationships likely exist with factors like IL-6 signalling: OR per SD CRP = 1.16 [95% CI: 1.09, 1.23], *P* = 7.23 × 10^−5^.

Whilst there was some evidence for a causal relationship between GGT and pneumonia susceptibility using an MR approach (supported five models being directionally consistent in GGT exerting an odds increasing effect on pneumonia, Fig. 4b, Supplementary Data 16), only the weighted mode and weighted median models were statistically significant – OR per SD GGT = 1.08 [95% CI: 1.02, 1.16], *P* = 0.01 (Weighted median). We therefore also considered a *cis*-acting model for GGT (the IV rs3859862 mapped to the *GGT1* gene) and observed only a non-significant trend in the same direction (*P* = 0.08). In summary, a causal relationship between GGT and pneumonia was suggested in an LCV model constructed using genome-wide SNP effects, which could support inhibition of this enzyme as a treatment target. With several GGT inhibitors under active development and under consideration for use in respiratory illness^43,44^; these data do marginally support this observation. However, given that this relationship was only weakly supported by MR, some caution and further investigation is warranted. We also compared the MR effect sizes to observational estimate of elevated GGT and lifetime pneumonia susceptibility in the UKBB cohort (Supplementary Methods). The observational association between GGT and pneumonia in the UKBB was directionally consistent and stronger than the MR estimate, with each standard deviation associated with a 13.54% [95% CI: 12.43%, 14.56%] increase in the odds of pneumonia amongst UKBB participants. Interestingly, this association was also significant amongst younger non-smoking females in the UK biobank at baseline (age < 45) who have relatively lower risk of pneumonia (OR = 1.354 [95% CI: 1.161, 1.553], *P* = 2.75 × 10^−5^. These variables are extremely heterogeneous and there are many potential confounders of the observed effect sizes, however, it supports the inferred relationship from the LCV construct, and to a lesser extent, some of the MR models.

### Precision treatment for pneumonia using pharmacologically orientated polygenic scoring

The pharmagenic enrichment score (PES) approach was applied to pneumonia susceptibility to identify drug repurposing candidates that could be targeted more precisely based on genetic risk^24,45,46^. Briefly, the PES is a genetic risk score specifically within a biological pathway that is targeted by a drug. The concept underlying the PES is that individuals with elevated genetic risk within a particular druggable set of genes may benefit from a pharmacological agent that modulates the pathway in question. Firstly, we sought to identify candidate pathways for PES in a hypothesis-free manner using the summary statistics from this study. 1030 gene-sets from the molecular signatures database (MSigDB) with a least one druggable gene were tested for an enrichment of the pneumonia common variant signal relative to all other genes at four different *P* value thresholds of variant inclusion (Online Methods, Supplementary Methods). The underlying concept of using *P* value thresholding in the competitive enrichment tests is that there may be different biological insights that can be gained by focusing on pathways enriched with variations at different levels of the polygenic signal. For example, consider a pathway which displays enrichment when all variants are considered (*P* < 1), versus a pathway which only displays enrichment when nominally significant variants are included (*P* < 0.05). The nominally significant variants represent a less ‘polygenic’ signal relative to all variants, and thus, may encompass distinct biological processes. We identified 13 candidate druggable pathways for which a PES could be constructed after applying FDR correction (FDR < 0.05) across all *P* thresholds considering either a conservative or liberal annotation boundary for SNPs to gene annotation (Supplementary Data 17 and 18). These were as follows: Cytokine-induced activation of matrix metalloproteinases (*P* = 2.95 × 10^−7^), Genes upregulated by IL6 and STAT3 signalling (*P* = 9.44 × 10^−7^), Complement cascade (*P* = 3.63 × 10^−6^), Metabolism of proteins (*P* = 1.03 × 10^−5^), Aldosterone-regulated sodium reabsorption (*P* = 1.33 × 10^−5^), Post-translational protein modification (*P* = 2.26 × 10^−5^), Notch-mediated HES/HEY network (*P* = 2.53 × 10^−5^), Expression of cyclins regulates progression through the cell cycle by activating cyclin-dependent kinases (*P* = 2.80 × 10^−5^), G_0_ and Early G_1_ cell cycle phase (*P* = 6.26 × 10^−5^), Regulation of retinoblastoma protein (*P* = 7.67 × 10^−5^), Cytokine–cytokine receptor interaction (P = 9.51 × 10^−5^), Regulation of transcriptional activity by PML (*P* = 1.23 × 10^−4^), and Jak-STAT signalling (*P* = 1.23 × 10^−4^). Of these sets, 10 were at least nominally significant across both genic boundary configurations, and thus, considered for further annotation. These gene-sets were then tested for an overrepresentation amongst drug targets within 85 anatomical therapeutic classification (ATC) level codes (*P* < 5.81 × 10^−4^. Supplementary Data 19). For instance, the aldosterone-regulated sodium reabsorption pathway was enriched for the targets of C03 (diuretic) and C01 (cardiac therapy) classified agents, whilst the cytokine–cytokine receptor interaction pathways had overrepresented targets of L03/L04 (immunostimulants/immunosuppressants) drugs and metabolism of proteins was overrepresented amongst antithrombotic agents (B01).

We then considered which of these candidate druggable gene-sets that displayed an enrichment of the genetic signal contained at least one of the two therapeutic targets we prioritised in this study (*MUC5AC* and *TNFRSF1A*). *MUC5AC* overlapped two gene-sets related to protein modification, in line with the known biology of mucins, metabolism of proteins and post-translational protein modification. *TNFRSF1A* was a member of four candidate PES, specifically—regulation of transcriptional activity by PML, genes upregulated by IL6 and STAT3 signalling, cytokine–cytokine receptor interaction, and cytokine-induced activation of matrix metalloproteinases. These PES were then profiled in the UKBB at the same *P* value threshold and genic boundary definition as a replication of their association with lifetime pneumonia susceptibility. We found that the association with pneumonia susceptibility with following two PES overlapping our prioritised genic targets nominally replicated in the UKBB (*N*_Cases_ = 15,158, *N*_Controls_ = 320,213): metabolism of proteins (OR per SD in PES = 1.032 [95% CI: 1.016, 1.049], *P* = 1.12 × 10^−4^, *N*_SNP_ = 410), and post-translational protein modification (OR per SD in PES = 1.029 [95% CI: 1.011, 1.043], *P* = 1.51 × 10^−3^, *N*_SNP_ = 320). As in previous work, we then included genome wide pneumonia polygenic risk score (PRS) as a covariate in the association model at the same *P* value threshold to ensure the signal for this gene-set was not purely driven by background inflation of genetic risk^45,46^. Both sets remained nominally significant after adjustment for total genome-wide pneumonia common variant risk in individuals (*P* < 0.05, *χ*^2^ test of residual deviance). We emphasise that these results only survive multiple-testing correction when the marginal PRS-unadjusted association is considered, however, nominal replication using PRS adjusted signal is still notable given the much lower effective pneumonia sample size in the UKBB than our discovery GWAS and the conservativeness of the PRS covariation approach. Both these PES were significantly correlated with genome-wide pneumonia PRS at the same-threshold, although both were relatively small in magnitude (*r* < 0.15), whilst the PES demonstrated high correlation with each other (*r* = 0.68), as would be expected given their overlapping biology. As a result, this reinforces that these pathways may be an identifiable component of the polygenic architecture of pneumonia that could be used to direct inhibitors of *MUC5AC* to patients with elevated pneumonia genetic risk amongst protein modification processes such as glycosylation. Moreover, the cytokine–cytokine receptor interaction PES that contained *TNFRSF1A* did display some nominal enrichment of the pneumonia signal in the UKBB cohort, however, at a different *P* value threshold than what was most significant in the summary data (UKBB: *P*_T_ = 0.005, *P* = 1.33 × 10^−3^, *N*_SNP_ = 44).

## Discussion

In this study, we uncovered association signals for lifetime pneumonia susceptibility outside of the MHC region. Interestingly, there was also a low frequency variant in the MHC itself which reached genome-wide significant that confers a relatively large (~65%) increase in the odds of pneumonia, reflecting the immense heterogeneity spanned within this region. Further analyses of the MHC signal are warranted, particularly to deconvolve specific HLA types that may contribute to pneumonia susceptibility and progression. We implemented an integrative gene prioritisation approach to identify plausible causal genes at each of the non-MHC loci, with the most consistent evidence found for *TNFRSF1A*, *IL6R*, and *MUC5AC*. *MUC5AC* is an interesting candidate given that it has been previously implicated in the pathogenesis of respiratory illness and the role of mucins in physical defence against pathogens via mucociliary clearance^47^. This heavily glycosylated protein is lowly expressed in normal respiratory epithelium, however, is upregulated upon in response to perturbagens, such as viral infection^48,49^. We posit that upregulation of *MUC5AC* may be deleterious in the context of pneumonia given recent evidence that this protein can enhance airway inflammation induced by viral infection^50^, although dissection of the mechanisms of variants in this locus are warranted. There are also some preliminary data that suggests *MUC5AC* is upregulated in the airway mucus of patients with severe COVID-19, although these studies were conducted using small sample sizes^51,52^. Interestingly, therapies specifically targeting mucin-linked *O*-glycosylation are now under active development, including a recently proposed hexosamine analogue that demonstrated potent inhibition of *O*-glycan biosynthesis and downregulation of neutrophil infiltration in rodents^53^. The prioritisation of *TNFRSF1A* as a risk gene for pneumonia susceptibility was also notable given the existence of pre-clinical compounds developed for its inhibition in acute respiratory distress syndrome. *TNFRSF1A* (TNFR1) is one of the two central cellular receptors of tumour necrosis factor-alpha, an important inflammatory cytokine induced during acute inflammation. Murine knock-out studies have demonstrated that selective inhibition of TNFR1 protects against sepsis and lung injury^19,54,55^, with this study providing genetic evidence for the first time that downregulation of this protein would be risk-decreasing for pneumonia.

A limitation in the use of GWAS signals to inform drug development or repurposing is that inter-individual heterogeneity may confound the effectiveness of these compounds^24^. In other words, the unique genetic risk factors carried by each individual diagnosed with a complex trait like pneumonia may implicate different underlying biological processes as of greater or lesser importance. Our group has previously developed the *pharmagenic enrichment score* (PES) to overcome this by targeting treatment to those with elevated genetic risk within pathways relevant to the drug. A hypothesis free scan of druggable pathways that could be used as PES implicated interesting clinically actionable biology in pneumonia susceptibility—for example, we found an enrichment of pneumonia susceptibility genetic risk within the aldosterone-regulated sodium reabsorption pathway. Aldosterone signalling is an important regulator of blood pressure, with hypertension shown to be a risk factor for incident pneumonia both observationally and through Mendelian randomisation^56^, with this PES potentially supporting precision indication of antihypertensive interventions. Some of the implicated pathways also overlapped *MUC5AC* and *TNFRSF1A*, the two most confident therapeutic targets identified in this study based on the individual genome-wide association signals. We also were able to replicate the overrepresentation of the pneumonia polygenic signal within protein modification pathways in which *MUC5AC* participates in the independent UKBB cohort, suggesting that a PES to direct mucin inhibitors may be particularly clinically tractable. Detailed discussion of the strengths and limitations of the PES methodology have been featured in previous publications^24,45,46^; however, further work is required to assess the suitability of the PES framework to direct treatment, such as clinical trials for compounds stratified by the PES to investigate their relevance to treatment response.

The genetic architecture of pneumonia susceptibility was revealed to exhibit non-zero genetic correlations with several clinically interesting traits, although the strength of the relationship with psychiatric phenotypes was particularly interesting given it was only marginally impacted by the possible confounders of differing pneumonia phenotype definitions or adjustment for genetically proxied smoking heaviness. There is a wealth of epidemiological data which have previously supported that several psychiatric illnesses increase the risk of pneumonia^57–60^, whilst infection and pneumonia is also a putative risk factor for subsequent mental illness^61–63^. For instance, a British registry study estimates that the rate ratio for incident pneumonia after hospitalisation for a range of psychiatric illnesses was consistently greater than two^57^. Additional study is required to fully understand what underlies the genetic correlation between pneumonia susceptibility and psychiatric illness, but we present some plausible hypotheses forthwith. Firstly, we found local significant positive correlations between the MHC pneumonia signal and psychiatric phenotypes like major depressive disorder. This may imply that MHC-related factors like HLA-types which increase the odds of pneumonia susceptibility may also be risk-increasing for psychiatric illness. Other inflammatory mediators outside of the MHC could also contribute to this shared genetic architecture throughout the rest of the genome. Our group has previously shown several psychiatric GWAS are significantly genetically correlated with various indices of leucocyte abundance^27^, suggesting pleiotropy with inflammatory biology. It is possible that there could be an influence of both aberrant inflammation and inability to respond adequately to infection in this relationship, however, the biological pathways that could underlie this remain uncharacterised. One interesting candidate for additional study would be tumour necrosis factor-alpha biology given elevated *TNFRSF1A* has also been associated with schizophrenia, bipolar disorder, and autism in the large post-mortem brain expression study from the PsychENCODE consortium^64^, although genetic evidence from GWAS for this gene is still lacking in the context of psychiatric phenotypes.

There are a number of important limitations that should be considered related to the pneumonia GWAS in this study. Firstly, this GWAS was conducted using samples from European ancestry. It will be critical to translate findings related to host-genetic influences on pneumonia that future efforts strive to collect trans-ancestral data, particularly due to concerns about the portability of European GWAS signals and the advantages in finemapping afforded by including multiple ancestries^65^. The SNP heritability for pneumonia derived in this study was also relatively low, and it remains unclear how heterogeneity amongst the phenotype definition of pneumonia may contribute to this. In other words, given that pneumonia is caused by a variety of factors and may go undiagnosed in some individuals, detailed phenotyping data would potentially assist in resolving the genetic architecture of this disorder. For example, a GWAS on susceptibility verses pneumonia severity will likely reveal some different biological insights. This could also be aided by stratified analyses by age, given pneumonia is more pervasive in the elderly. However, an advantage of this study relative to recent GWAS of COVID-19 severity is the larger number of cases and a more generalised phenotype that could be applicable to a variety of pneumonia aetiologies. The putative therapeutic candidates suggested in this study must also be viewed in light of the low heritability of pneumonia and the need for clinical validation. Despite these challenges, we believe that further resolving the host-genetic architecture of pneumonia will be invaluable to public health efforts to more effectively prevent and manage this illness.

## Methods

### Genome-wide meta-analysis of pneumonia

This research used GWAS summary statistics that received relevant ethics approval. The use of UK Biobank data individual level data in that portion of the study was approved by the UK Biobank Access Committee (application ID = 58432). The genome-wide meta-analysis was performed using two primary study cohorts from 23andMe, Inc. and FinnGen (release 6), respectively, with full details of these cohorts and the meta-analysis procedure detailed in the supplementary methods. Summary statistics for a self-reported pneumonia phenotype were obtained from 23andMe as outlined by Tian et al.^5^. This self-reported phenotype was derived from an online survey of 23andMe customers about their medical history. In the final 23andMe GWAS after quality control (QC), there were 40600 cases and 90039 controls. In addition, summary statistics for pneumonia were downloaded from the sixth release of the FinnGen database which combines genotype data from Finnish biobanks and digital health record data from Finnish health registries. The pneumonia phenotype chosen was All pneumoniae (J10 pneumonia), for which 33723 cases and 226682 controls were available for GWAS after QC.

The 23andMe and FinnGen summary statistics were meta-analysed using an inverse-variance weighted model with fixed effects as implemented by METAL version March 2011^66^. Firstly, we meta-analysed common variants, defined as sites with allele frequency > 1% in both the 23andMe and FinnGen cohorts. Variants were retained if they were available in both summary statistics and had an imputation quality that exceeded 0.6 for variants not physically genotyped, resulting in 6897087 common sites with an effect size estimate from the meta-analysis and a total sample size of 391044 individuals. Imputed rare variants available in both studies (INFO > 0.6) were also subjected to meta-analysis, with 882364 low frequency variants considered. In both instances, we further tested for heterogeneity between the contributing studies using Cochran’s *Q* test. Genome-wide summary statistics from the IVW meta-analysis were processed using the FUMA v1.3.7 (Functional Mapping and Annotation of Genome-Wide Association Studies) platform^67^. Genome-wide significant variants were characterised using the traditional *P* < 5 × 10^−8^ threshold, whilst suggestive significance was defined using a more lenient threshold of *P* < 1 × 10^−5^. We utilised the default settings for defining independent significant SNPs (*r*^2^ ≤ 0.6) and lead SNPs (*r*^2^ ≤ 0.1). The reference panel population for LD estimation was the 1000 genomes phase III European reference panel, with LD blocks within 250 kb of each other merged into a single locus. We examined the effect of conditioning on two smoking GWAS via the multi-trait-based conditional & joint analysis (mtCOJO) framework implemented in GCTA v 1.93.2 beta (Supplementary Methods)^8,68^. For the common lead SNP, we additionally performed a phenome-wide association study using the IEUGWAS database version 3.7.0 (https://gwas.mrcieu.ac.uk/), reporting SNPs using a conventional phenome-wide significance threshold of *P* < 1 × 10^−5^. Given the most significant association in this database for the *MUC5AC* lead SNP was a GWAS of adult-onset asthma^69^, we also tested whether the association of SNPs proximal to *MUC5AC* was driven by the same underlying causal variant, assuming a single causal variant, via the *coloc* colocalisation methodology implemented in version 4 of the package^70^. We also sought to replicate our results in two UK biobank (UKBB) pneumonia GWAS, specifically, a self-reported pneumonia phenotype performed in the automated GWAS pipeline by the MRC IEU group (ukb-b-4533, https://gwas.mrcieu.ac.uk/datasets/ukb-b-4533/), as well as a phecode ICD-10 UKBB GWAS performed in an automated series of GWAS by the authors of the SAIGE methodology (https://pheweb.org/UKB-SAIGE/pheno/480)^71^.

### Estimation of SNP-based heritability

SNP based heritability was computed using LD score regression (LDSR) with 1000 genomes phase 3 European LD scores and weights^25^. We converted the heritability estimate to the liability scale assuming the population prevalence of pneumonia as that of pneumonia in the FinnGen dataset (12.96%), as well as a more conservative estimate based on ICD-10 diagnosed pneumonia in the UK biobank (UKBB) sample (3.20% - Supplementary Methods). Genetic correlation between the self-reported and clinically ascertained pneumonia input GWAS was estimated using ‘munged’ variants aligned to the non-MHC HapMap3 reference panel via LDSR.

### Prioritisation of plausible genes from genome-wide significant loci

We implemented an integrative approach to priortise causal associations at each of the genome-wide significant loci outside of the MHC region. Firstly, we annotated the closet gene to the lead SNP using the Open Targets genetics platform (v2022.22)^13^. FUMA was then utilised to annotate all SNPs within each loci with eQTLs to identify the gene for which the strongest eQTL signal could be identified, with annotations sourced from a variety of databases including GTEx and the eQTL catalogue, as outlined elsewhere^67^. Similarly, pQTLs were annotated using the Open Targets platform. ANNOVAR, as implemented by FUMA, annotated any variants as non-synonymous^72^. We then finemapped each of these regions to map genes to the derived credible sets. SNP-effect sizes were finemapped using method which leverages asymptotic Bayes’ factors (ABF) to estimate credible sets under the assumption of a single causal variant^73^. Specifically, we utilised Wakefield’s method to approximate ABFs assuming a prior variance of 0.2^2^, which reflects the belief that the confidence intervals of estimated variant effect sizes expressed as odds ratios ranging from around 0.68 to 1.48. Given that the posterior probability for causality of each variant is proportional to its Bayes’ factor, these can be summed until a prespecified probability (*ρ*) is reached, thus, constituting a *ρ* set of putative causal variants. In this study, we derived 95% credible sets. A single causal variant was assumed such that we did not have to account for LD between variants, which has been demonstrated to be problematic in finemapping studies which prespecify more than one causal variant using references external to the GWAS like the 1000 genomes project panel^74^. Secondly, these regions were finemapped using marginal TWAS *Z* scores for each gene within and proximal to the locus via FOCUS v0.6.10^12^. We utilised the default Bernoulli prior (*p* = 1 × 10^−3^) and chi-square prior variance (*nσ*^2^ = 40) to approximate Bayes’ factors for each gene, and thus, derive the posterior inclusion probabilities (*PIP*) for each gene to be causal given its observed TWAS *Z*. Two integrative scoring pipelines were also considered to priortise genes—the *variant to gene* (V2G) approach implemented by Open Targets for the lead SNP and the RegulomeDB rank^13,15^. The V2G scoring metric integrates information related to physical distance, eQTL (eGene) and pQTL (pGene) annotation, epigenetic annotation with data like promoter capture Hi-C, and in silico functional prediction. RegulomeDB was implemented by FUMA and applies a rank score to each SNP in the locus leveraging eQTL annotation, in silico transcription factor binding prediction, and chromatin data, with SNPs ranked as “1” more likely to impact binding and expression of a linked target. We identified the gene with the highest V2G for the lead SNP and the gene mapped to the lowest RegulomeDB rank considering all SNPs in the locus. Finally, we also annotated all variants in each implicated region with an in silico predicted scaled combined annotation dependent depletion (CADD) score via FUMA, with the gene mapped to the highest scoring SNP prioritised^14^. In summary, we identified the gene/s which satisfied any of the following for the four non-MHC loci: closet gene to lead SNP, most significant eGene and pGene, gene with a non-synonymous variant, genes mapped to variants in the ABF 95% credible set, genes prioritised as causal by FOCUS, the highest V2G score for each lead SNP, the gene mapped to the variant with the lowest RegulomeDB rank, and the gene mapped to the variant with the highest CADD score.

### Phenome-wide consequences of genetically proxied *TNFRSF1A* inhibition

We sought to further investigate the utility of inhibiting *TNFRSF1A* as a treatment opportunity for pneumonia. A blood eQTL SNP from the eQTL catalogue with high posterior inclusion probability over 95% for that locus (rs1800692) was selected as an instrumental variable for an MR-pheWAS using the IEUGWAS database and FinnGen release 6^75^. We filtered the IEUGWAS database to retain GWAS catalogue imports, UKBB GWAS conducted by IEUGWAS, IEUGWAS curated consortia GWAS, UKBB brain imaging GWAS, and immune/metabolite GWAS (IEUGWAS codes = ukb-b, met-a, met-b, met-c, met-d, ubm-a, ebi-a, ieu-a, ieu-b). The effect of *TNFRSF1A* expression on each outcome GWAS was calculated using the Mendelian randomisation Wald ratio (ratio of coefficients) method^76,77^. We flipped the effect size directions of the Wald ratio results such that the beta represented the effect of decreased *TNFRSF1A* expression on the outcome phenotypes.

### Gene-based and gene-set association

Common variant (MAF > 0.01) SNP-wise *P* values were aggregated at gene-level using MAGMA v1.09^20^, using the 1000 genomes phase III European reference panel to approximate LD for the calculation of the test statistic. We considered three different strategies to annotate SNPs to genes for gene-based association testing; specifically, mapping SNPs only within the defined coordinates of genes, a conservative extension of genic boundaries to capture regulatory variation (5 kilobase (kb) upstream and 1.5 kb downstream), and a more liberal genic boundary extension (35 kb upstream, 10 kb downstream). The Bonferroni threshold for genic association was *P* < 2.68 × 10^−6^, accounting for the number of genes tested. Moreover, gene-based *P* values were leveraged for gene-set association using 21,765 gene-sets assembled by the g:Profiler platform^78^, and available at all three annotation boundary configurations. We meta-analysed the gene-set association *P* values from each boundary configuration via the Aggregated Cauchy Association Test (ACAT) (Supplementary Methods). Code for the Cauchy approach was obtained from (https://github.com/yaowuliu/ACAT) and outlined by Liu and Xie^21,79^. Moreover, a TWAS of pneumonia was performed using the FUSION package^22^. We utilised GTEx v7 SNP weights from three tissues that would be plausibly involved in the pathophysiology of pneumonia (whole blood, lung, and spleen). In addition, we probabilistically finemapped transcriptome-wide significant regions using the FOCUS as outlined above.

### Genetic correlation and causal inference

We estimated genetic correlation between pneumonia and 674 UK Biobank (UKBB) GWAS with a trait *h*^2^_SNP_ *Z* score > 4 from the Neale group (http://www.nealelab.is/uk-biobank) using LDSR. Genetic correlation was also applied to 9 psychiatric GWAS from the psychiatric genomics consortium with more curated phenotypes^28–35^, as well as a general cognitive ability GWAS^36^. Local genetic correlation estimates for the MHC region were obtained using the LAVA approach, ensuring that the traits considered (pneumonia, PTSD, ADHD, and MDD) all had significantly non-zero univariable heritability in this region^37^. For Bonferroni significant genetic correlation estimates, we constructed a LCV model to evaluate evidence for partial genetic causality between traits^26,27,46^. A strong estimate of the posterior genetic causality proportion (GCP) was defined as significantly different from zero (one sided *t*-test) and an absolute GCP estimate > 0.6. Weak GCP estimates close to zero for genetically correlated traits imply that their relationship is potentially mediated by horizontal pleiotropy, whereby there are shared pathways, but the two traits do not likely exhibit vertical pleiotropy by acting within the same pathway. In addition, we followed up the two biochemical traits (CRP and GGT) that exhibited evidence for partial genetic causality on pneumonia from the LCV model using two-sample Mendelian randomisation, as described in the supplementary methods.

### The *pharmagenic enrichment score* for precision drug repurposing

The PES framework to prioritise treatments for individuals using polygenic scores specifically partitioned into pathways or gene-sets with known drug targets^24,45,46^. Specifically, we identify druggable pathways with an enrichment of common variant associations relative to the rest of the genes tested and construct pathway-based risk scores for these gene-sets (Supplementary Methods). We investigated overrepresentation of ATC drug categories amongst these gene-sets using the GREP tool^80^. PES gene-sets that survived multiple-testing correction were then further considered to identify those overlapping the target genes prioritised in this study, *MUC5AC* and *TNFRSF1A*. We utilised the UK biobank (UKBB) cohort to replicate the association between these pneumonia PES that overlapped one of those target genes and lifetime pneumonia susceptibility^81,82^. These analyses are described in detail in the supplementary methods. The use of these data were approved by the UKBB access management services system (project ID: 58432). Briefly, we retained 336,896 unrelated white British ancestry participants and 13,568,914 autosomal variants that survived a series of quality control steps, including, imputation quality filtering (INFO > 0.8), MAF > 1 × 10^−4^, call rate > 0.98, and filtering strong deviations from the Hardy–Weinberg equilibrium. Self-reported pneumonia diagnosis and ICD-10 codes from hospital inpatient records were used to construct the pneumonia phenotype (Supplementary Methods). There were 15,138 individuals from the genotyped subset of the cohort included in the PES calculation with a primary or secondary diagnosis using the ICD-10 primary or secondary diagnosis codes relevant to pneumonia, or a self-reported pneumonia diagnosis at any assessment centre visit. In turn, there were 320,213 controls without a self-reported or clinically ascertained pneumonia diagnosis. SNPs were annotated to PES gene-sets using the same boundary configuration that was most significant from the summary statistics analyses (liberal or conservative), as well the best performing *P* value threshold for clumping and thresholding (C+T). Sample code for the implementation of the PES approach from our group can be found elsewhere—https://github.com/Williamreay/Pharmagenic_enrichment_score; The PES calculation was performed using the PRSice-2 v2.3.5 (linux)^83^. Firstly, we tested the baseline (marginal) association of these PES with pneumonia susceptibility using binomial logistic regression covaried for age, sex, the first 20 SNP derived principal components, and genotyping batch. Thereafter, we included genome-wide PRS at the same *P* value threshold as an additional covariate to account for the background genetic signal and tested for the significance of the PES coefficient using a *χ*^2^ test of residual deviance.

### Reporting summary

Further information on research design is available in the Nature Research Reporting Summary linked to this article.

## Supplementary information

Supplementary Information

Description of Additional Supplementary Files

Supplementary Data 1-11

Supplementary Data 12-19

Reporting Summary

## Data Availability

The top 10,000 SNPs from our meta-analysis have been deposited in the following GitHub repository. (https://github.com/Williamreay/Pneumonia_meta_GWAS/tree/master/Summary_statistics). This file contains the top 10,000 SNPs ranked in terms of their statistical significance. The full GWAS summary statistics for the 23andMe discovery data set will be made available through 23andMe to qualified researchers under an agreement with 23andMe that protects the privacy of the 23andMe participants. Researchers wishing to recapitulate our meta-analysis can apply for access for the 23andMe subset of the study (https://research.23andme.com/dataset-access/), and then meta-analyse with FinnGen release 6 summary statistics (https://r6.finngen.fi/) as described in our manuscript. The UK Biobank data can be obtained by approved researchers after direct application to the UK Biobank (https://www.ukbiobank.ac.uk/enable-your-research/apply-for-access). Additional GWAS summary statistics for other in traits of this study were obtained from the IEU open GWAS project (https://gwas.mrcieu.ac.uk/), the Psychiatric Genomics Consortium (https://www.med.unc.edu/pgc/download-results/), and the Neale Group (http://www.nealelab.is/uk-biobank).

## Code availability

The code used in our study is available on Github which can be found via the following link: https://github.com/Williamreay/Pneumonia_meta_GWAS. The code version used in this study is registered^84^.
