# Supplementary material for: The genetic architecture of pneumonia susceptibility implicates mucin biology and a relationship with psychiatric illness
Source: Nat Commun. 2022 Jun 29;13:3756. doi: 10.1038/s41467-022-31473-3 (PMC9243103; doi:10.1038/s41467-022-31473-3)
Supplement: Supplementary file 1 — Supplementary Information [file 41467_2022_31473_MOESM1_ESM.pdf]

The genetic architecture of pneumonia susceptibility implicates mucin biology and a relationship with psychiatric illness

SUPPLEMENTARY RESULTS

Heterogeneity amongst SNP-pneumonia effect sizes between cohorts in the munged summary statistics

As visualised below in supplementary figure 1, we found that considering the pneumonia summary statistics 'munged' to the HapMap3 panel, there were only a small number of variants that displayed suggestive association with pneumonia ($P < 1 \times 10^{-5}$) and significant heterogeneity.

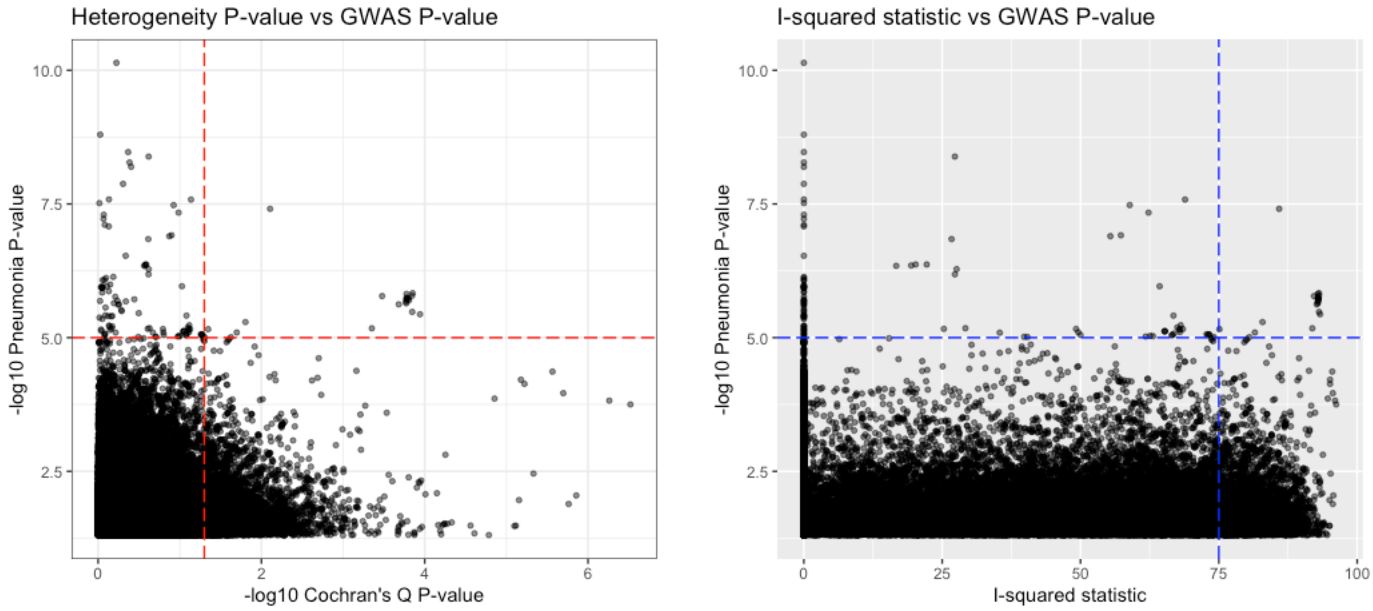

Supplementary Figure 1. Genome-wide tests of heterogeneity of SNP pneumonia-effect sizes between the 23andMe and FinnGen cohorts. We plot the two related metrics of heterogeneity (-$\log_{10}$ Cochran's Q P value and I^2 statistic) verses -$\log_{10}$ SNP-pneumonia GWAS P values for variants in the munged summary statistics (merged to the non-MHC HapMap3 panel) which displayed at least a nominal association with pneumonia ($P < 0.05$). We denote SNPs with suggestive significance ($P < 1 \times 10^{-5}$) on both plots with the horizontal

dotted line and nominal evidence of heterogeneity (left plot: Cochran's Q $P < 0.05$, right plot: $I^2 > 75$).

Quantile-quantile plot of test statistic inflation in the meta-analysis

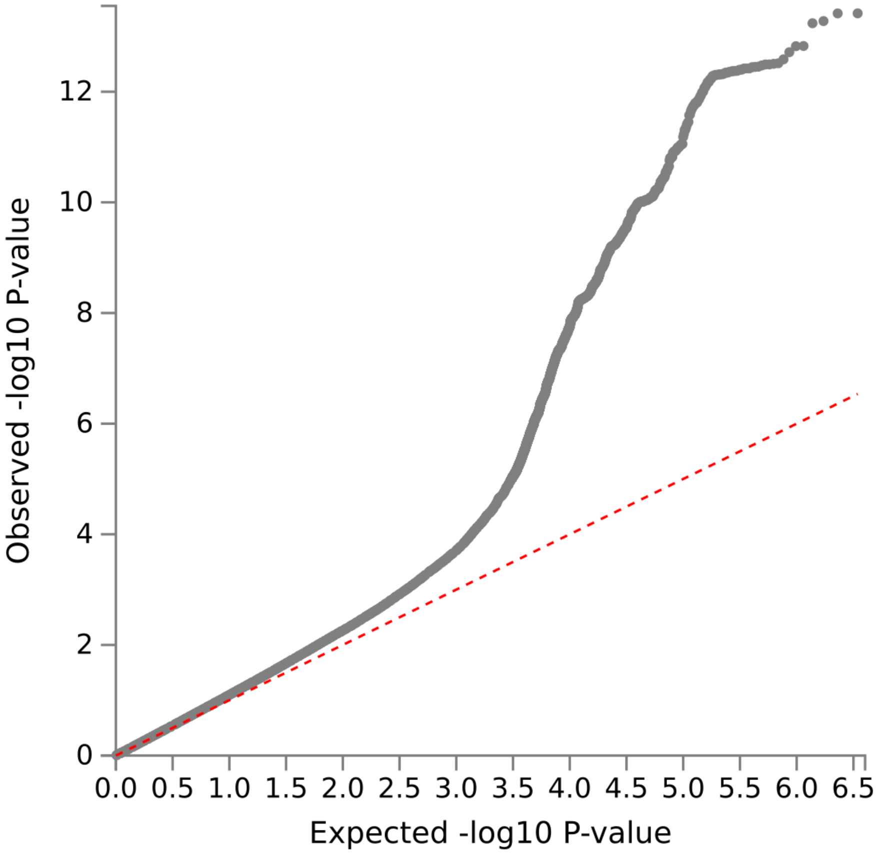

Supplementary Figure 2. Quantile-quantile (QQ) plot of common variant GWAS. Quantile-quantile plot which visualises expected versus observed association (two-sided P value), deviations from the diagonal line can be considered evidence of test-statistic inflation.

Colocalisation analyses of the mucin locus between adult-onset asthma and pneumonia susceptibility

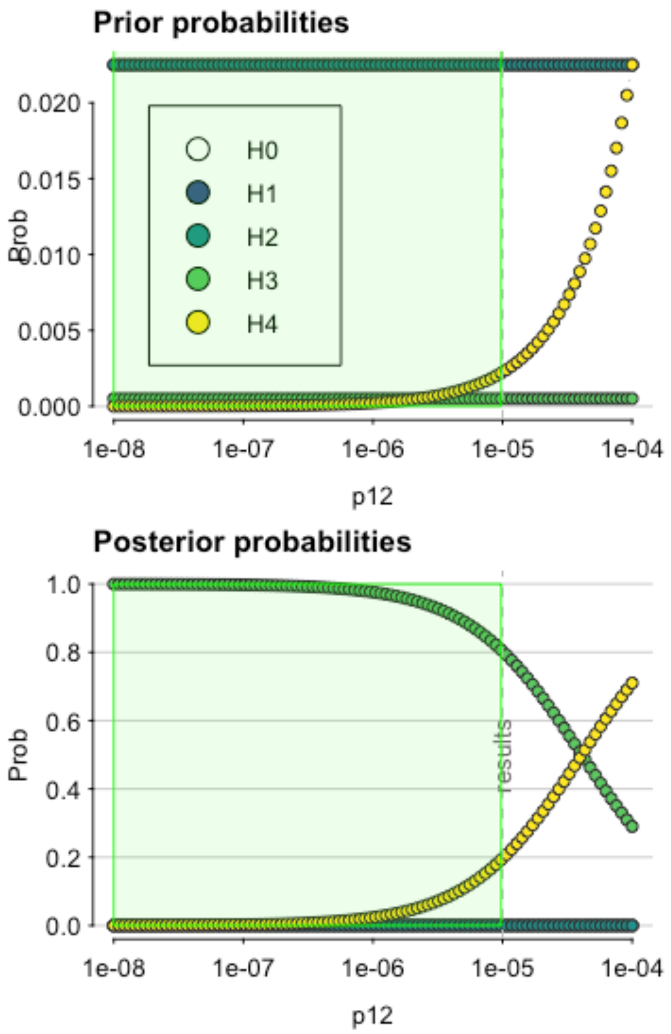

Supplementary Figure 3. Sensitivity of the posterior probability of a shared causal variant for adult-onset asthma and pneumonia in the mucin locus to different priors. We visualise the effect of using the default prior probability of hypothesis 4 (H_4, shared causal variant), which was 1 x 10^{5} and demonstrate strong evidence that the locus was associated with both traits but with a different underlying causal variant (H_3)– posterior probability > 90%. However, if a less conservative prior for H_4 was utilised, for example, < 1 x 10^{-4} , lowers the posterior probability of H_3 and raises the posterior probability of H_4.

MR-Clust mixture modelling results for the CRP to pneumonia model using a cluster inclusion probability threshold of 80%

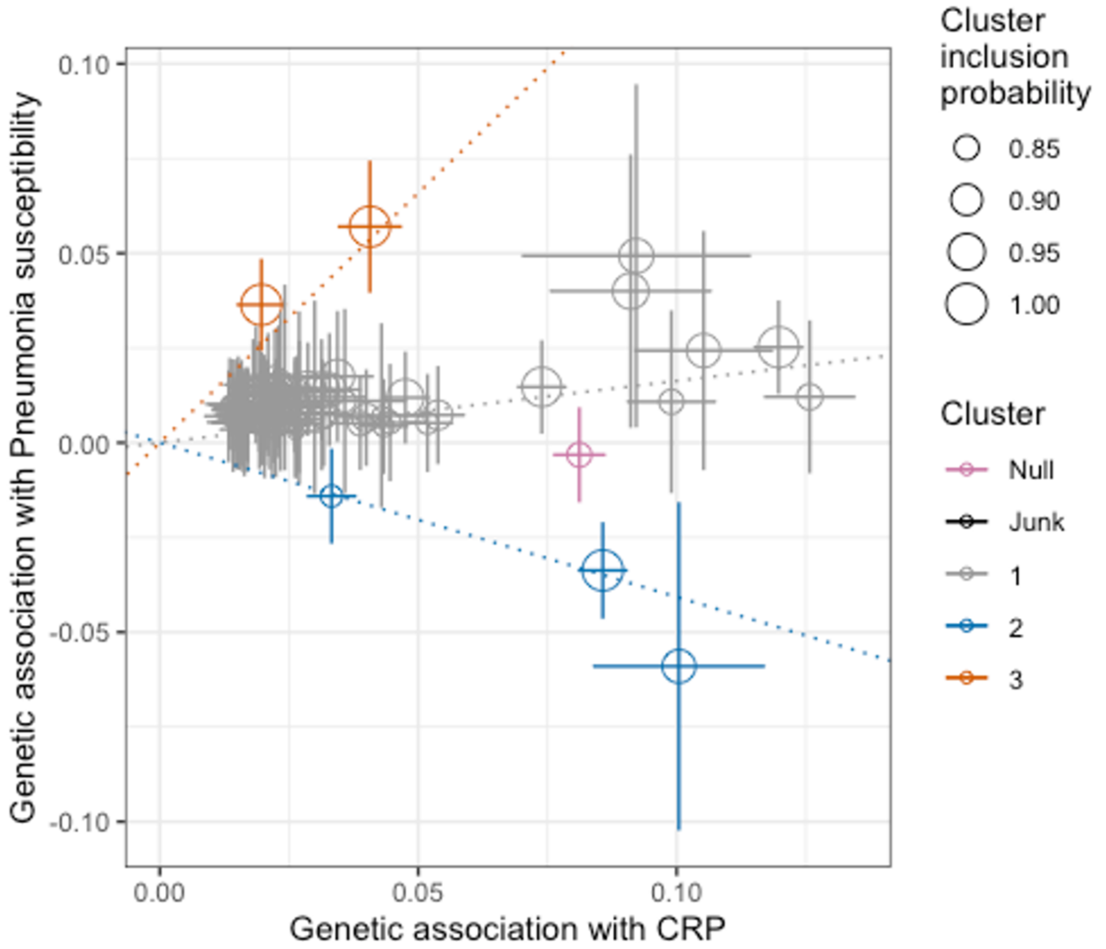

Supplementary Figure 4. Output of the MR-Clust approach for the effect of CRP on pneumonia susceptibility using a cluster allocation threshold of 80% for filtering instrumental variable SNPs. This is a mixture model framework that seeks to identify clusters of instrumental variables with similar causal estimates. The size of the point denotes the cluster inclusion probability, relating to the conditional probability of cluster membership. The null cluster, coloured pink, relates to IVs with null effect, whilst the black "junk cluster" are variants that were not parsimoniously assigned to any cluster. The three different non-null or junk clusters are each coloured grey, orange, and blue, respectively, with a trend line indicative of the mean cluster effect. The sample size of the CRP GWAS was 343,524. The error bars denote the standard error estimates of the Wald Ratio for each instrumental variable.

SUPPLEMENTARY METHODS

The GWAS meta-analysis was performed using two primary study cohorts from 23andMe Inc. and FinnGen (release 6), respectively, as described below.

<u>23andMe</u>

Summary statistics for a self-reported pneumonia phenotype were obtained from 23andMe as outlined by Tian *et al.* [1]. This self-reported phenotype was derived from an online survey of 23andMe customers about their medical history; specifically, the following two questions: "*Have you ever been diagnosed by a doctor with any of the following infectious conditions?" (Pneumonia: Yes, No, I don't know)*; *"Have you ever been diagnosed by a doctor with any of the following infectious conditions?"/"Pneumonia" (Never, 1-2 times, 3-5 times, More than 5 times, I'm not sure*). The endpoint for GWAS was binary such that individuals who answered yes to the first question, or at least once for the latter question, were cases, and those answering no or never, respectively, were controls. Moreover, cases could not have a negative response to one question and positive to the other, whilst controls were not permitted to have any positive responses. In the final GWAS after quality control (QC), there were 40600 cases and 90039 controls. The majority of self-reported pneumonia cases were female (56%), conversely, the majority of controls were male (54%). Participant age was described by four bins in the 23andMe meta-data for this GWAS: under 30, 30-45, 45-60, and over 60, with the oldest category the most common for cases (41%) and controls (31%). All individuals included in the 23andMe analyses provided informed consent and answered surveys online according to their human subjects protocol, which was reviewed and approved by Ethical & Independent Review Services, a private institutional review board (http://www.eandireview.com).

DNA was extracted from saliva samples and genotyped using one of four genotyping platforms, as described previously [1]. This GWAS was restricted to unrelated individuals with European ancestry [1,2], with ancestry assigned by the *Ancestry composition* pipeline and relatedness tested through identity-by-descent (IBD) estimation [3]. Imputation and phasing were performed separately for the four different genotyping platforms, with Beagle v3.3.1 utilised to phase the samples and Minimac2 leveraged for imputation to the March 2012 version three release of the 1000 genomes reference panel.

Genetic association in the 23andMe sample was performed using logistic regression covaried for age, sex, and the first four SNP derived principal components. This model assumed additive effects and *P* values were computed using a likelihood ratio test. Imputed dosages were utilised as opposed to 'best-guess' genotypes. Variants outside the pseudo-autosomal region on the X chromosome were coded as homozygous diploid for males. In our study, we retained only SNPs for meta-analysis that passed internal QC – specifically, SNPs were flagged and not retained if any of the following were met: *P* value for deviation from the Hardy-Weinberg equilibrium (HWE) of $P < 10^{-20}$, call rate < 90%, SNPs with a significant date of genotype effect (ANOVA of SNPs by a factor binning genotype data into 20 roughly equal bins), mitochondrial and Y chromosome SNPs, SNPs genotyped only on the first genotyping platform, an average imputation $R^2 < 0.5$ or a minimum $R^2 < 0.3$, and SNPs indicative of an imputation batch effect ($P < 10^{-50}$, ANOVA by a factor of imputation batch). We further filtered variants with an average imputation $R^2 > 0.6$ for consistency with FinnGen release 6.

FinnGen (release six)

Summary statistics for pneumonia were downloaded from the sixth release of the FinnGen database which combines genotype data from Finnish biobanks and digital health record data from Finnish health registries. The pneumonia phenotype chosen was *All pneumoniae* (J10 pneumonia), for which 33723 cases and 226682 controls were available for GWAS after QC. This phenotype is level C in the ICD-hierarchy and encompasses several endpoint definitions which are as follows: viral pneumonia (Inflammation of the lung parenchyma that is caused by a viral infection) - Hospital Discharge registry/Cause of Death registry: ICD-10: J12, ICD-9: 480, ICD-8: 480; Pneumonia due to *Streptococcus pneumoniae* (streptococcal pneumonia: A febrile disease caused by streptococcus pneumoniae) - Hospital Discharge registry/Cause of Death registry: ICD-10: J13, ICD-9: 481, ICD-8: 48199; Pneumonia due to *Haemophilus influenzae* (bacterial pneumonia: Inflammation of the lung parenchyma that is caused by bacterial infections) - Hospital Discharge registry/Cause of Death registry: ICD-10: J14, ICD-8: 48210; Bacterial pneumonia, not elsewhere classified - Hospital Discharge registry/Cause of Death registry: ICD-10: J15, ICD-9: 482, ICD-8: 482; Pneumonia due to other infectious organisms, not elsewhere classified - Hospital Discharge registry/Cause of Death registry: I CD-10: J16, ICD-9: 483, ICD-8: 48399; Pneumonia, organism unspecified - Hospital Discharge registry/Cause of Death registry: ICD-10: J18, ICD-9: 485, ICD-8: 4850[2-9]|486. In the entire FinnGen cohort, the unadjusted prevalence of the *All pneumoniae* phenotype is 12.96%, with a mean age at first event of 54.33 and an absolute risk of fatality at five years of

3%. The three drug classes most likely to be purchased after this diagnosis for the cohort were penicillins, fluroquinolones, and anilides.

The full genotyping, imputation, and GWAS protocol for release three of FinnGen has been outlined previously (https://finngen.gitbook.io/documentation/). Briefly, individuals from FinnGen contributing biobanks or cohorts were genotyped via Illumina or Affymetrix chip arrays. Individuals with sex mismatch, missigness > 5%, excess heterozygosity and non-Finish ancestry were removed. The final cohort consisted of unrelated, Finish ancestry individuals over the age of 18. Before imputation variants with call rate < 98%, a P value for deviation from HWE < $1x10^{-6}$, and minor allele count (MAC) < 3 were discarded, and pre-phasing performed using Eagle 2.3.5. Genotype imputation was undertaken using Beagle 4.1 and the population-specific SISu v3 reference panel that consists of 3775 high coverage whole genome-sequencing samples from six Finnish cohorts. The GWAS utilised the SAIGE model, which builds on the concept of logistic mixed models, covaried for age, sex, the first ten SNP-derived principal components, and genotyping batch [4]. Association testing was performed for variants with a minimum MAC of 5.

Definition of a genome-wide significant locus

Genome-wide summary statistics from the IVW meta-analysis were processed using the FUMA v1.3.6 (Functional Mapping and Annotation of Genome-Wide Association Studies) platform [5]. Genome-wide significant variants were characterised using the traditional $P < 5 \times 10^{-8}$ threshold, whilst suggestive significance was defined a more lenient threshold of $P < 1x10^{-5}$. We used the default settings for defining independent significant SNPs ($r^2 \leq 0.6$), followed by lead SNPs ($r^2 \leq 0.1$). The reference panel population for LD estimation was the 1000 genomes phase 3 European cohort, with LD blocks within 250 kb of each other merged into a single locus.

Estimation of SNP-based heritability

SNP based heritability was computed using LD score regression (LDSR) with 1000 genomes phase 3 LD scores and weights [6]. We converted the heritability estimate to the liability scale assuming the population prevalence of pneumonia of that of pneumonia in the FinnGen dataset (12.61%), as well as a more conservative population prevalence based on ICD-10 diagnosed pneumonia in the UK biobank (3.20% - see section: Pneumonia phenotype definition in the UK biobank cohort).

Finemapping of genome-wide significant loci

We finemapped the three-novel genome-wide significant loci outside of the MHC region by using a method which leverages approximated asymptotic Bayes' factors (ABF) to estimate credible sets under the assumption of a single causal variant [7,8]. Specifically, we utilised Wakefield's method to approximate Bayes' factors assuming a prior variance of 0.2^2, which reflects the belief that the confidence intervals of estimated variant effect sizes expressed as odds ratios range from around 0.68 to 1.48. Given that the posterior probability for causality of each variant is proportional to its Bayes' factor, these can be summed until a prespecified probability (ρ) is reached, thus constituting a 'ρ x 100%' set of putative causal variants.

Impact of smoking and smoking heaviness on genetic associations with pneumonia

We wished to investigate the potential confounding influence of genetic associations with smoking behaviour on the pneumonia genome-wide meta-analysis by conditioning the GWAS on two smoking related GWAS using the multi-trait-based conditional and joint analysis (mtCOJO) method [9]. The smoking phenotypes selected were lifetime smoking initiation (ever vs never smoked, N = 262990) and smoking heaviness (cigarettes per day, N = 263954) [10]. We recalculated heritability for the pneumonia summary statistics conditioned on lifetime smoking initiation and smoking heaviness separately, assuming the same population prevalence for liability scale conversion.

Gene-based and gene-set association

Common variant (MAF > 0.01) SNP-wise *P* values were aggregated at gene-level using MAGMA v1.09b [11]. Gene-based *P* values were calculated using the *snp-wise=mean* MAGMA model, whereby the test statistic is the sum of squared SNP-wise *Z* scores, with $Z \sim \mathcal{N}(0, S)$, given *S* is a matrix of correlations between SNPs. Gene coordinates in hg19 assembly were obtained from NCBI and the 1000 genomes phase 3 European panel utilised as an LD reference. Genes within the MHC region were not considered due to the haplotype complexity of that region, as is usual practice. We firstly annotated SNPs to genes using the defined genic coordinates, as well as two windows to extend the genic boundaries (conservative - 5kb upstream and 1.5 kb downstream, liberal - 35kb upstream and 10 kb downstream) to capture potential regulatory variation. The Bonferroni threshold for genic association was $P < 2.68 \times 10^{-6}$, accounting for the number of genes tested. Moreover, gene-based *P* values for annotation boundary configuration separately were leveraged for gene-set association using 21,765 gene-

sets from a range of ontological databases aggregated by the g:Profiler platform. After probit transformation of *P* to *Z*, a linear regression model was constructed such that *Z* was the outcome and binary indicator of gene-set membership an explanatory variable to test whether genes in the set were more associated than all other genes considered. This model was covaried for confounders including gene-size and MAC, as described elsewhere [11,12]. To boost power for gene-set association, we meta-analysed the set-wise *P* values at each boundary configuration (no boundary, conservative, and liberal) using the Aggregated Cauchy Association Test (ACAT). Code for the Cauchy combination test was obtained from (https://github.com/yaowuliu/ACAT). The test statistic (*T*) is a sum of *P*-values (p_i) transformed to approximate a Cauchy distribution, which is also flexible to incorporate weights (w_i; equation 1), however, no weighting was applied in this instance and all *P* values had equal weight.

$$T = \sum_{i=1}^{k} w_i \tan\{(0.5 - p_i)\pi\} \quad (1)$$

Due to the heavy tail of the Cauchy distribution, *T* is insensitive to correlations amongst the *P* values that arise due to these *P* values being from the same sample, with the combined meta-analytic *P* value approximated using the cumulative density function of the Cauchy distribution (equation 2). We corrected for multiple testing across all meta-analytic gene-set association *P* values using Benjamini-Hochberg (FDR) correction, with FDR < 0.05 set as the threshold and FDR < 0.1 as a suggestive cut-off.

$$P_{\text{Combined}} \approx \frac{1}{2} - \left[\frac{\left\{\arctan\left(\frac{T}{w}\right)\right\}}{\pi}\right] \quad (2)$$

Transcriptome-wide association studies of pneumonia

A transcriptome-wide association study (TWAS) of pneumonia was performed using the FUSION method to identify novel associations beyond genome-wide significant loci [13]. TWAS tests the association between genetic variants comprising the models of predicted gene expression and the phenotype of interest. SNP weights were derived for genes with a significant contribution of *cis* acting SNPs to expression variability (*cis*-h^2 $P < 0.01$) using lung, whole blood, and spleen RNAseq GTEx v7 data. Another Bayesian method FOCUS was then utilised to finemap the TWAS associations [14]. Given observed TWAS statistics, the marginal posterior inclusion probability (*PIP*) was calculated and subsequently used to compute a credible set

with 90% probability (ρ) of containing the causal gene ($c_i = 1$). As FOCUS allows the null model to be predicted as a possible member of the credible set, we excluded any genes for which that occurred. The credible set (S) was defined by summing normalised PIP such that ρ was exceeded, sorting the genes and then including those genes until at least ρ of the normalized-posterior mass is explained (equation three).

$$S\{Gene_1, \ldots, Gene_k\} = \sum_{i=1}^{k} PIP\ (c_i = 1 | Z_{TWAS})\ \geq\ \rho \quad (3)$$

The Bernoulli prior for each causal indicator was set as the default $p = 1 \times 10^{-3}$, with a default prior variance for effects at causal genes set as 40 ($n\sigma_c^2 = 40$). Previous work has demonstrated that FOCUS computed PIPs were robust to different specified prior variances [14]. In all instances, we utilised a multi-tissue panel obtained from FOCUS GitHub repository which combines GTEx v7 SNP-weights with other FUSION TWAS weights (https://github.com/bogdanlab/focus/wiki, GTEx v7 with METSIM, CMC, YFS, and NTR).

Genetic correlation and causal inference

Bivariate linkage disequilibrium score regression (LDSR) was performed between pneumonia and a variety of UKBB phenotypes subjected to GWAS by the Neale group. Summary statistics from the pneumonia meta-analysis were cleaned ('munged') prior to LDSR using munge_sumstats.py and merged with common HapMap3 SNPs excluding the major histocompatibility complex (MHC) region due to its LD complexity, as is usual practice [6]. For Bonferroni significant genetic correlation estimates, we constructed a latent causal variable (LCV) model to evaluate evidence for partial genetic causality between traits. The RunLCV.R and MomentFunctions.R scripts were leveraged to perform these analyses (https://github.com/lukejoconnor/LCV). The LCV framework assumes that a latent variable, L, mediates the genetic correlation between two traits (trait one, trait two), and uses the mixed fourth moments of the bivariate effect size distribution to estimate the mean posterior genetic causality proportion (GCP) as described in detail by O'Connor and Price [15]. The GCP estimate quantifies the magnitude of genetic causality between the two traits. GCP values range from −1 to 1 (full genetic causality), within these limits positive values indicate greater partial genetic causality of trait one on two, and vice versa for negative values. All traits were munged prior to LCV analyses, with only HapMap3 SNPs (MAF > 0.05) outside the MHC region retained in accordance with the LDSR analyses. We utilised the baseline 1000 genomes phase 3 LD scores for HapMap3 SNPs (MHC excluded). A two-sided t test was used to assess

whether the estimated GCP was significantly different from zero, with an absolute posterior mean GCP > 0.6 considered a reliable estimate.

Mendelian randomisation (MR) was then implemented to further probe the relationships between C-reactive protein (CRP) and pneumonia, and gamma glutamyl-transferase (GGT) and pneumonia. These traits are ideally suited for MR as these are continuous exposures with characterised biology to assess instrumental variable (IV) validity. The primary model was an inverse-variance weighted (IVW) estimator [16], with a comparison made between a model with fixed effects versus multiplicative random effects. The random effects approach is better suited to instances with IV exposure-outcome effect heterogeneity, as was tested with Cochran's Q test [17]. The IVW model is considered to be the most well-powered out of the suite of MR methods we implemented in this study, however, it makes a potentially unrealistic assumption that all IVs are valid. The use of a genetic variant as an IV is underpinned by three central assumptions, as has been discussed extensively elsewhere [18,19]:

IV1: the variant is rigorously associated with the exposure;

IV2: the variant is independent of all confounders of the exposure-outcome relationship ;and,

IV3: the variant is associated with the outcome only by acting through the exposure (independent conditional on the exposure and confounders).

It should be noted that only the first assumption can be directly tested, as a result, we utilised four additional MR methods as a sensitivity analysis that make different underlying assumptions about IV validity. Firstly, a weighted median estimator that is subject to the 'majority valid' assumption (unbiased estimate as long as < 50% of IVs are invalid) by taking the median of ratio estimates rather than the mean like the IVW method [18]. Secondly, a mode-based estimator was implemented that is underpinned by the related 'plurality valid' assumption [20]. Thirdly, an MR-Egger model was constructed which is an adaption of Egger regression wherein the exposure effect is regressed against the outcome with an intercept term added to represent the average pleiotropic effect [19]. Finally, we utilised the outlier robust MR-Pleiotropy Residual Sum and Outlier (MR-PRESSO) framework [21]. The advantage of this method is that it seeks to identify outlier IVs by considering the residual sum of squares (RSS, a heterogeneity measure of IV estimates) and testing for deviations from a simulated Gaussian distribution of expected RSS when each IV is iteratively removed. We note that no outliers were identified in the triglyceride or GGT model. Three additional statistical methods were implemented to test for evidence of potential confounding pleiotropy: testing whether the

Egger intercept is significantly different from zero, the MR-PRESSO global pleiotropy test, and a leave-one-out analysis; whereby each IV was iteratively removed and the IVW estimate recalculated to evaluate evidence of disproportionately large IV effects that may be indicative of a pleiotropic signal. It is of critical importance that researchers interpreting the output of these statistical methods to evaluate pleiotropy are cognisant that it is not possible to exclude a confounding influence of horizontal pleiotropy through statistical methods alone. The mixture model approach MR-Clust was also applied to the CRP to pneumonia model, as well as replication using a non-UKBB GWAS of CRP.

Moreover, we investigated the observational association between GGT concentration amongst individuals from the UK biobank without missing values for the respective biochemical trait or covariate values. This was done given the relationship between GGT and pneumonia has not been well observationally characterised, unlike the in the case of CRP. The definition of the pneumonia phenotype in the UK biobank is discussed in the subsequent section of this document (*PES analyses in the UK biobank*). The measures of GGT as baseline was scaled to have zero mean and unit variance. We constructed a binomial logistic regression model to test the association between GGT and pneumonia, covaried for sex, age, age^2, sex*age, sex*age^2, smoking status (ever vs never smoked), and Townsend deprivation index. Furthermore, we also tested the association between individuals in the 90th percentile for each measure and pneumonia diagnosis relative to the remaining participants. The final sample size was 466,435 for the GGT analyses. Furthermore, we repeated the test between GGT levels and pneumonia diagnosis amongst a subset of the UK biobank cohort who have relatively lower risk of pneumonia, specifically, females aged under 45 at the time of assessment who are self-reported lifetime non-smokers. The sample size was 14,947 for the GGT cohort, with the logistic regression model here still covaried for Townsend deprivation index.

The pharmagenic enrichment score (PES) framework

The *pharmagenic enrichment score* (PES) is an approach to perform genomics-informed precision drug repurposing or indication of newly discovered drugs [22]. Specifically, pathways (gene-sets) with known drug targets are identified which display some evidence of harbouring an enrichment of trait associated variation relative to all other genes. These pathways then become the basis for a genetic risk score using only those variants mapped to genes within the pathway (PES). Individuals with high genetic risk within a particular druggable pathway (elevated PES) may benefit from a drug which modulates that pathway. We identified

candidate gene-sets to construct pneumonia PES by using a modified version of MAGMA. We implemented P value thresholding in the calculation of the gene-based test statistic, as we have described previously[22–24]. Briefly, the underlying concept of this is that there may be different biological insights that can be gained by focusing on pathways enriched with variations at different levels of the polygenic signal. For example, consider a pathway which displays enrichment when all variants are considered ($P < 1$), versus a pathway which only displays enrichment when nominally significant variants are included ($P < 0.05$). The nominally significant variants represent a less 'polygenic' signal relative to all variants, and thus, may encompass distinct biological processes. We posit that this concept is somewhat analogous to the P value thresholding when selecting the most parsimonious PRS configuration, whereby different P value thresholds seem to fit better depending on the target phenotype of interest. In accordance with our previous work we somewhat arbitrarily selected four P value thresholds which balance capturing different elements of the polygenic signal with encompassing enough SNPs to construct scores using variants only within a biological pathway, which may not be realistic at lower P thresholds – specifically, we chose, $P < 1$ (all SNPs), $P < 0.5$, $P < 0.05$, and $P < 0.005$ [22]. The definition of a 'druggable' biological pathway has been described elsewhere [12], briefly the MSigDB gene-sets used for gene-set association in this study were processed to identify a subset of gene-sets (N=1030) with at least one high confidence gene targeted by an approved pharmacological agent.

We selected pathways that survived multiple-testing correction for an enrichment of pneumonia associated variation relative to all other genes at that threshold by applying correction via the Benjamini-Hochberg (BH) method (FDR < 0.05) to all thresholds combined. These associations can be interpreted based on the P value threshold for the model, for example, at gene-set which survives FDR correction that includes only variants which displayed a nominally significant univariable association with pneumonia ($P < 0.05$) is indicative of a set of genes that are more associated with pneumonia than all other genes with at least one SNP that had $P < 0.05$ in the GWAS. The BH approach was implemented rather than Bonferroni as several gene-sets will be tested multiple times at different P-value thresholds, and thus, the assumption of independence underlying Bonferroni correction likely means this would be overly conservative. We conducted these analyses using conservative and liberal genic boundaries, and only retained gene-sets that survived multiple testing correction in one configuration and were at least nominally significant ($P < 0.05$) in the other. In

particular, we focused on the subset of these sets which overlapped *MUC5AC* and *TNFRSF1A*, given these were two confident therapeutic targets prioritised in this study.

Pneumonia phenotype definition in the UK biobank cohort

We utilised the UK biobank (UKBB) cohort to test the association between a pneumonia PRS and pneumonia phenotypes recorded for these participants. The UKBB is a large, longitudinal study that has recorded extensive phenotypic information accompanied by genetic data for the majority of individuals [25]. Individuals for whom the following information was non-missing were retained (N = 498,989) – phenotypic sex (field 31.0.0), age at assessment (field 21003.0.0), self-reported smoking history (ever vs never smoked, field 20160), and the Townsend deprivation index metric (field 189.0.0).

There were two main criteria used to define our set of lifetime-pneumonia cases that used either self-reported or hospital inpatient records. Firstly, we identified individuals who self-reported a pneumonia diagnosis at any of the assessment visits. Secondly, we leveraged primary and secondary ICD-10 codes for each participant from hospital inpatient records. The ICD-10 codes used to define the pneumonia phenotype were as follows: J100 – influenza with pneumonia, influenza virus identified, J110 – influenza with pneumonia, virus not identified, J12 – viral pneumonia, not elsewhere classified, J13 - Pneumonia due to *Streptococcus pneumoniae*, J14 - Pneumonia due to *Haemophilus influenzae*, J15 - Bacterial pneumonia, not elsewhere classified, J16 - Pneumonia due to other infectious organisms, not elsewhere classified, J17 - Pneumonia in diseases classified elsewhere, and J18 - Pneumonia, organism unspecified. Pneumonia cases were individuals with a relevant ICD-10 code or a self-reported lifetime pneumonia diagnosis (N = 22,312).

Genotyping, imputation, and quality control of the UK biobank SNP array data

The genotyping and imputation procedures for UKBB participants have been described extensively elsewhere [26]. We obtained chromosome-wise imputed data in Oxford bgen format from the UKBB as per our application (version 3 imputation) and restricted variants to sites in the Haplotype Reference Consortium panel (~40 million variants). Sample exclusions comprised of any individuals who satisfied one or more of the following criteria – missing sex recorded at baseline (field 31), mismatch between recorded sex and genetically inferred sex (field 22001), evidence of sex chromosome aneuploidy (field 22019), excess heterozygosity and missing rate (field 22027), ten or more third-degree relatives identified in the sample (field

22021), exclusion from the kinship inference process (field 22021), and other flagged sample exclusions in the UKBB meta-data (field 22010), . Thereafter, unrelated individuals were retained by virtue of being in the principal components analysis (PCA) conducted by the UKBB (as these individuals were deemed unrelated by the UKBB). The analyses in this manuscript were restricted to a homogeneous white-British subset of the UKBB to attempt to guard against unwanted effects of population stratification. This subset was ascertained by selecting those participants with self-identified 'white British' ancestry and a very similar genetic ancestry based on the projection of eigenvectors from the PCA in the work done by the UKBB [26].

Post-imputation QC was as follows and performed using PLINK 2 – firstly, well-imputed variants were retained using a threshold of variant INFO > 0.8, followed by excluding variants that satisfied or more of the following: MAF < 1×10^{-4}, strong deviation from the Hardy-Weinberg equilibrium ($P < 1 \times 10^{-10}$) and call rate < 0.98. This resulted in a final set of 336,896 participants and 13,568,914 variants that survived all of the above QC. We reperformed PCA using this filtered white British ancestry subset using FlashPCA2 v2.0 [27] in order to calculate eigenvectors to include as covariates in downstream analyses. As is usual practice, we only included variants with MAF > 0.05 in relative linkage equilibrium (pairwise $r^2 < 0.05$), that were physically genotyped on both array types and were not in regions of long-range LD known to confound PCA, such as the MHC region on chromosome six [28].

Generation of the PES and PRS profiles

We excluded the MHC region from the PES and genome wide PRS due to the complexity of this region, as is usual practice. Variants were mapped to each gene comprising the PES pathway if they were within the defined gene coordinates, or in the genic boundary used for the identification of the PES gene-sets. PRSice-2 v2.3.5 (linux) was used to calculate the scores for biallelic autosomal variants. The *P* value threshold for variant inclusion in the PES was the same as the threshold most significant for the pathway in the gene-set association. We utilised the default LD r^2 for PRSice-2 ($r^2 < 0.1$), with the PES calculated under an additive model whereby the effect size of each independent variant in the pathway was multiplied by its zygosity and summed for each individual. There were 15,138 individuals classified as a pneumonia case (self-reported or ICD-10) in the genotyped European UKBB subset after QC, and 320,213 controls. We tested the baseline (marginal) association of these PES with pneumonia susceptibility using binomial logistic regression covaried for age, sex, the first 20 SNP derived principal components, and genotyping batch. We then included genome-wide

PRS at the same P value threshold as an additional covariate and tested for the significant of the PES coefficient using a χ^2 test of residual deviance.

Operating systems and software versions

The unix scripts and command line inputs for this manuscript were run using either macOS Catalina version 10.15.4 or Ubuntu 18.04.5 LTS. R scripts were computed using R version 3.6.0, whilst python scripts were computed using python 3.8.3. The scripts utilised for this study will be available on GitHub: https://github.com/Williamreay/Pneumonia_meta_GWAS

SUPPLEMENTARY REFERENCES

1. Tian, C. *et al.* Genome-wide association and HLA region fine-mapping studies identify susceptibility loci for multiple common infections. *Nat Commun* **8**, 599 (2017).
2. Durand, E. Y., Do, C. B., Mountain, J. L. & Macpherson, J. M. *Ancestry Composition: A Novel, Efficient Pipeline for Ancestry Deconvolution*. http://biorxiv.org/lookup/doi/10.1101/010512 (2014) doi:10.1101/010512.
3. Henn, B. M. *et al.* Cryptic distant relatives are common in both isolated and cosmopolitan genetic samples. *PLoS ONE* **7**, e34267 (2012).
4. Zhou, W. *et al.* Efficiently controlling for case-control imbalance and sample relatedness in large-scale genetic association studies. *Nat. Genet.* **50**, 1335–1341 (2018).
5. Watanabe, K., Taskesen, E., van Bochoven, A. & Posthuma, D. Functional mapping and annotation of genetic associations with FUMA. *Nat Commun* **8**, 1826 (2017).
6. Bulik-Sullivan, B. *et al.* An atlas of genetic correlations across human diseases and traits. *Nat. Genet.* **47**, 1236–1241 (2015).
7. Wellcome Trust Case Control Consortium *et al.* Bayesian refinement of association signals for 14 loci in 3 common diseases. *Nat. Genet.* **44**, 1294–1301 (2012).
8. Wakefield, J. Bayes factors for genome-wide association studies: comparison with P-values. *Genet Epidemiol* **33**, 79–86 (2009).

9. Zhu, Z. *et al.* Causal associations between risk factors and common diseases inferred from GWAS summary data. *Nat Commun* **9**, 224 (2018).
10. Liu, M. *et al.* Association studies of up to 1.2 million individuals yield new insights into the genetic etiology of tobacco and alcohol use. *Nat. Genet.* **51**, 237–244 (2019).
11. de Leeuw, C. A., Mooij, J. M., Heskes, T. & Posthuma, D. MAGMA: generalized gene-set analysis of GWAS data. *PLoS Comput. Biol.* **11**, e1004219 (2015).
12. Reay, W. R. & Cairns, M. J. Pairwise common variant meta-analyses of schizophrenia with other psychiatric disorders reveals shared and distinct gene and gene-set associations. *Transl Psychiatry* **10**, 134 (2020).
13. Gusev, A. *et al.* Integrative approaches for large-scale transcriptome-wide association studies. *Nat. Genet.* **48**, 245–252 (2016).
14. Mancuso, N. *et al.* Probabilistic fine-mapping of transcriptome-wide association studies. *Nat. Genet.* **51**, 675–682 (2019).
15. O'Connor, L. J. & Price, A. L. Distinguishing genetic correlation from causation across 52 diseases and complex traits. *Nat. Genet.* **50**, 1728–1734 (2018).
16. Burgess, S., Butterworth, A. & Thompson, S. G. Mendelian randomization analysis with multiple genetic variants using summarized data. *Genet. Epidemiol.* **37**, 658–665 (2013).
17. Bowden, J., Hemani, G. & Davey Smith, G. Invited Commentary: Detecting Individual and Global Horizontal Pleiotropy in Mendelian Randomization-A Job for the Humble Heterogeneity Statistic? *Am J Epidemiol* **187**, 2681–2685 (2018).
18. Bowden, J., Davey Smith, G., Haycock, P. C. & Burgess, S. Consistent Estimation in Mendelian Randomization with Some Invalid Instruments Using a Weighted Median Estimator. *Genet. Epidemiol.* **40**, 304–314 (2016).

19. Bowden, J., Davey Smith, G. & Burgess, S. Mendelian randomization with invalid instruments: effect estimation and bias detection through Egger regression. *Int J Epidemiol* **44**, 512–525 (2015).

20. Hartwig, F. P., Davey Smith, G. & Bowden, J. Robust inference in summary data Mendelian randomization via the zero modal pleiotropy assumption. *Int J Epidemiol* **46**, 1985–1998 (2017).

21. Verbanck, M., Chen, C.-Y., Neale, B. & Do, R. Detection of widespread horizontal pleiotropy in causal relationships inferred from Mendelian randomization between complex traits and diseases. *Nat. Genet.* **50**, 693–698 (2018).

22. Reay, W. R., Atkins, J. R., Carr, V. J., Green, M. J. & Cairns, M. J. Pharmacological enrichment of polygenic risk for precision medicine in complex disorders. *Sci Rep* **10**, 879 (2020).

23. Reay, W. R. *et al.* Genetic association and causal inference converge on hyperglycaemia as a modifiable factor to improve lung function. *eLife* **10**, e63115 (2021).

24. Reay, W. R. & Cairns, M. J. Advancing the use of genome-wide association studies for drug repurposing. *Nat Rev Genet* (2021) doi:10.1038/s41576-021-00387-z.

25. Sudlow, C. *et al.* UK biobank: an open access resource for identifying the causes of a wide range of complex diseases of middle and old age. *PLoS Med* **12**, e1001779 (2015).

26. Bycroft, C. *et al.* The UK Biobank resource with deep phenotyping and genomic data. *Nature* **562**, 203–209 (2018).

27. Abraham, G., Qiu, Y. & Inouye, M. FlashPCA2: principal component analysis of Biobank-scale genotype datasets. *Bioinformatics* **33**, 2776–2778 (2017).

28. Price, A. L. *et al.* Long-range LD can confound genome scans in admixed populations. *Am. J. Hum. Genet.* **83**, 132–135; author reply 135-139 (2008).
