# Supplementary material for: The genetic architecture of pneumonia susceptibility implicates mucin biology and a relationship with psychiatric illness
Source: Nat Commun. 2022 Jun 29;13:3756. doi: 10.1038/s41467-022-31473-3 (PMC9243103; doi:10.1038/s41467-022-31473-3)
Supplement: Supplementary file 2 — Description of Additional Supplementary Files [file 41467_2022_31473_MOESM2_ESM.pdf]

Description of Additional Supplementary Files

File Name: Supplementary Data 1
Description: Phenome-wide association study (pheWAS) of rs11245979 in the IEUGWAS database

File Name: Supplementary Data 2
Description: Phenome-wide association study (pheWAS) of rs4149581 in the IEUGWAS database

File Name: Supplementary Data 3
Description:. Phenome-wide association study (pheWAS) of rs9283753 in the IEUGWAS database

File Name: Supplementary Data 4
Description:. Phenome-wide association study (pheWAS) of rs6684439 in the IEUGWAS database

File Name: Supplementary Data 5
Description: Annotated rare variants associated with pneumonia susceptibility (P < 1e-05)

File Name: Supplementary Data 6
Description: Phenome-wide Mendelian randomisation (ieugwas db) - blood *TNFRSF1A* expression

File Name: Supplementary Data 7
Description: Phenome-wide Mendelian randomisation (FinnGen r6) - blood *TNFRSF1A* expression

File Name: Supplementary Data 8
Description: MAGMA gene-based association (no genic boundary extension)

File Name: Supplementary Data 9
Description: MAGMA gene-based association (5 kb upstream, 1.5 kb downstream genic boundary extension)

File Name: Supplementary Data 10
Description: MAGMA gene-based association (35 kb upstream, 10 kb downstream genic boundary extension)

File Name: Supplementary Data 11
Description: MAGMA gene-set association results - Cauchy meta-analytic P across three genic boundary configurations (FDR < 0.1)

File Name: Supplementary Data 12
Description: Transcriptome-wide association study of pneumonia susceptibility (GTEx blood, lung, spleen)

File Name: Supplementary Data 13
Description: Genetic correlation (LDSR) estimates between pneumonia susceptibility and Neale group automated GWAS (h2 Z > 4)

File Name: Supplementary Data 14
Description: Genetic correlation estimates between curated psychiatric GWAS and pneumonia suscpebility using different phenotype definitions

File Name: Supplementary Data 15

Description: CRP to pneumonia Mendelian randomisation estimates

File Name: Supplementary Data 16
Description: GGT to pneumonia Mendelian randomisation estimates

File Name: Supplementary Data 17
Description: Candidate gene-sets for PES gene-sets enriched with the pneumonia susceptibility common variant signal at different *P* value thresholds (conservative genic boundaries)

File Name: Supplementary Data 18
Description: Candidate gene-sets for PES enriched with the pneumonia susceptibility common variant signal at different *P* value thresholds (liberal genic boundaries)

File Name: Supplementary Data 19
Description: Candidate PES gene-sets enriched for an ATC level 2 code category after multiple-testing correction (GREP)
