# Supplementary material for: The genetic architecture of pneumonia susceptibility implicates mucin biology and a relationship with psychiatric illness
Source: Nat Commun. 2022 Jun 29;13:3756. doi: 10.1038/s41467-022-31473-3 (PMC9243103; doi:10.1038/s41467-022-31473-3)
Supplement: Supplementary file 5 — Reporting Summary [file 41467_2022_31473_MOESM5_ESM.pdf]

nature portfolio

Corresponding author(s): Murray Cairns

Last updated by author(s): Jun 2, 2022

Reporting Summary

Nature Portfolio wishes to improve the reproducibility of the work that we publish. This form provides structure for consistency and transparency in reporting. For further information on Nature Portfolio policies, see our Editorial Policies and the Editorial Policy Checklist.

Statistics

For all statistical analyses, confirm that the following items are present in the figure legend, table legend, main text, or Methods section.

n/a	Confirmed	
☐	☒	The exact sample size (*n*) for each experimental group/condition, given as a discrete number and unit of measurement
☐	☒	A statement on whether measurements were taken from distinct samples or whether the same sample was measured repeatedly
☐	☒	The statistical test(s) used AND whether they are one- or two-sided<br>*Only common tests should be described solely by name; describe more complex techniques in the Methods section.*
☐	☒	A description of all covariates tested
☐	☒	A description of any assumptions or corrections, such as tests of normality and adjustment for multiple comparisons
☐	☒	A full description of the statistical parameters including central tendency (e.g. means) or other basic estimates (e.g. regression coefficient) AND variation (e.g. standard deviation) or associated estimates of uncertainty (e.g. confidence intervals)
☐	☒	For null hypothesis testing, the test statistic (e.g. *F*, *t*, *r*) with confidence intervals, effect sizes, degrees of freedom and *P* value noted<br>*Give P values as exact values whenever suitable.*
☐	☒	For Bayesian analysis, information on the choice of priors and Markov chain Monte Carlo settings
☒	☐	For hierarchical and complex designs, identification of the appropriate level for tests and full reporting of outcomes
☒	☐	Estimates of effect sizes (e.g. Cohen's *d*, Pearson's *r*), indicating how they were calculated

Our web collection on statistics for biologists contains articles on many of the points above.

Software and code

Policy information about availability of computer code

Data collection	No software was used for data collection.
Data analysis	Code utilised in this study is available on GitHub - https://github.com/Williamreay/Pneumonia_meta_GWAS. The following software packages and tools were used for data analysis: METAL v March 2011 (https://github.com/statgen/METAL), FUMA v1.3.7 (https://fuma.ctglab.nl/), GCTA v 1.93.2 (https://yanglab.westlake.edu.cn/software/gcta/#Overview), IEUGWAS database version 3.7.0 (https://gwas.mrcieu.ac.uk/), ldsc v1.0.1 (https://github.com/bulik/ldsc), Open Targets Platform v2022.22 (https://www.opentargets.org/), FOCUS v0.6.10 (https://github.com/bogdanlab/focus), MAGMA v1.09 (https://ctg.cncr.nl/software/magma), FUSION v Nov 2021 (https://github.com/gusevlab/fusion_twas), LCV v 1 June 2020 (https://github.com/lukejoconnor/LCV), pharmagenic enrichment score v August 26 2021 (https://github.com/Williamreay/Pharmagenic_enrichment_score), PRSice-2 v2.3.5 (linux) (https://www.prsice.info/).

For manuscripts utilizing custom algorithms or software that are central to the research but not yet described in published literature, software must be made available to editors and reviewers. We strongly encourage code deposition in a community repository (e.g. GitHub). See the Nature Portfolio guidelines for submitting code & software for further information.

Data

Policy information about availability of data

All manuscripts must include a data availability statement. This statement should provide the following information, where applicable:

- Accession codes, unique identifiers, or web links for publicly available datasets
- A description of any restrictions on data availability
- For clinical datasets or third party data, please ensure that the statement adheres to our policy

The top 10,000 SNPs from our meta-analysis have been deposited in the following GitHub repository. (https://github.com/Williamreay/Pneumonia_meta_GWAS/

tree/master/Summary_statistics).. This file contains the top 10,000 SNPs ranked in terms of their statistical significance. The full GWAS summary statistics for the 23andMe discovery data set will be made available through 23andMe to qualified researchers under an agreement with 23andMe that protects the privacy of the 23andMe participants. Researchers wishing to recapitulate our meta-analysis can apply for access for the 23andMe subset of the study (https://research.23andme.com/dataset-access/), and then meta-analyse with FinnGen release 6 summary statistics (https://r6.finngen.fi/) as described in our manuscript. The UK Biobank data can be obtained by approved researchers after direct application to the UK Biobank (https://www.ukbiobank.ac.uk/enable-your-research/apply-for-access). Additional GWAS summary statistics for other in traits this study were obtained from the IEU open GWAS project (https://gwas.mrcieu.ac.uk/), the Psychiatric Genomics Consortium (https://www.med.unc.edu/pgc/download-results/), and the Neale Group (http://www.nealelab.is/uk-biobank).

Field-specific reporting

Please select the one below that is the best fit for your research. If you are not sure, read the appropriate sections before making your selection.

☒ Life sciences ☐ Behavioural & social sciences ☐ Ecological, evolutionary & environmental sciences

For a reference copy of the document with all sections, see nature.com/documents/nr-reporting-summary-flat.pdf

Life sciences study design

All studies must disclose on these points even when the disclosure is negative.

Sample size

Sample size was not predetermined, the largest sample size data available at time of analysis was utilised in all instances.

Data exclusions

Genetic variants removed before meta-analysis if the imputation quality (INFO) was less than 0.6. In the UK Biobank, We obtained chromosome-wise imputed data in Oxford bgen format from the UKBB as per our application (version 3 imputation) and restricted variants to sites in the Haplotype Reference Consortium panel (~40 million variants). Sample exclusions comprised of any individuals who satisfied one or more of the following criteria – missing sex recorded at baseline (field 31), mismatch between recorded sex and genetically inferred sex (field 22001), evidence of sex chromosome aneuploidy (field 22019), excess heterozygosity and missing rate (field 22027), ten or more third-degree relatives identified in the sample (field 22021), exclusion from the kinship inference process (field 22021), and other flagged sample exclusions in the UKBB meta-data (field 22010), . Thereafter, unrelated individuals were retained by virtue of being in the principal components analysis (PCA) conducted by the UKBB (as these individuals were deemed unrelated by the UKBB). The analyses in this manuscript were restricted to a homogeneous white-British subset of the UKBB to attempt to guard against unwanted effects of population stratification. This subset was ascertained by selecting those participants with self-identified 'white British' ancestry and a very similar genetic ancestry based on the projection of eigenvectors from the PCA in the work done by the UKBB 25.

Post-imputation QC was as follows and performed using PLINK 2 – firstly, well-imputed variants were retained using a threshold of variant INFO > 0.8, followed by excluding variants that satisfied or more of the following: MAF < 1x10-4, strong deviation from the Hardy-Weinberg equilibrium (P < 1 x 10-10) and call rate < 0.98. This resulted in a final set of 336,896 participants and 13,568,914 variants that survived all of the above QC. We reperformed PCA using this filtered white British ancestry subset using FlashPCA2 v2.0 in order to calculate eigenvectors to include as covariates in downstream analyses. As is usual practice, we only included variants with MAF > 0.05 in relative linkage equilibrium (pairwise r2 < 0.05), that were physically genotyped on both array types and were not in regions of long-range LD known to confound PCA, such as the MHC region on chromosome six.

Replication

We attempted to replicate our findings in independent GWAS as fully described in the methods – specifically, we utilised two automated GWAS that encompassed a self-reported pneumonia phenotype (NCase = 6572, NControls = 456,361) and ICD-10 derived pneumonia diagnoses (NCase = 10,059, NControls = 398,538). In the self-reported pneumonia UKBB GWAS, we found that no SNPs replicated at genome-wide significance, however, three of the lead SNPs for the chromosome 1, 5, and 11 loci were nominally associated in the same direction (rs6684439: P = 7.78 x 10-3; rs11245979: P =0.04; rs9283753: P = 0.045), The ICD-10 phenotype GWAS in the UKBB did not replicate any of our non-MHC genome-wide significant SNPs at even nominal significance, although there was trend for rs4149581 (P = 0.074).

Randomization

This was an observational genetic study designed to test for an association of genetic factors with lifetime pneumonia susceptibility, therefore, randomization would not have been feasible or appropriate in this study design.

Blinding

Blinding was not applicable or necessary in GWAS analyses.

Reporting for specific materials, systems and methods

We require information from authors about some types of materials, experimental systems and methods used in many studies. Here, indicate whether each material, system or method listed is relevant to your study. If you are not sure if a list item applies to your research, read the appropriate section before selecting a response.

Materials & experimental systems

n/a	Involved in the study
☒	☐ Antibodies
☒	☐ Eukaryotic cell lines
☒	☐ Palaeontology and archaeology
☒	☐ Animals and other organisms
☐	☒ Human research participants
☒	☐ Clinical data
☒	☐ Dual use research of concern

Methods

n/a	Involved in the study
☒	☐ ChIP-seq
☒	☐ Flow cytometry
☒	☐ MRI-based neuroimaging

Human research participants

Policy information about studies involving human research participants

Population characteristics	In the entire FinnGen cohort, the unadjusted prevalence of the All pneumoniae phenotype is 12.96%, with a mean age at first event of 54.33 and an absolute risk of fatality at five years of 3%. The entire FinnGen cohort was 66% male and 44% female . 23andMe: The majority of self-reported pneumonia cases were female (56%), conversely, the majority of controls were male (54%). Participant age was described by four bins in the 23andMe meta-data for this GWAS: under 30, 30-45, 45-60, and over 60, with the oldest category the most common for cases (41%) and controls (31%). In the UKBB cohort with genetic data considered in this study 54% were female with a mean age of 57 at time of enrolment.
Recruitment	23andMe: self-report questionnaire by 23andMe customers. FinnGen: clinically ascertained from ICD-10 records. UK Biobank: Recruited from the general population through advertisement.
Ethics oversight	The UK Biobank analysis were approved by the access committee (ID:58432)

Note that full information on the approval of the study protocol must also be provided in the manuscript.
